# CrkII/Abl phosphorylation cascade is critical for NLRC4 inflammasome activity and is blocked by *Pseudomonas aeruginosa* ExoT

**DOI:** 10.1038/s41467-022-28967-5

**Published:** 2022-03-11

**Authors:** Mohamed F. Mohamed, Kajal Gupta, Josef W. Goldufsky, Ruchi Roy, Lauren T. Callaghan, Dawn M. Wetzel, Timothy M. Kuzel, Jochen Reiser, Sasha H. Shafikhani

**Affiliations:** 1 Department of Medicine, Rush University Medical Center, Chicago, IL USA; 2 Division of Hematology/Oncology/Cell Therapy, Rush University Medical Center, Chicago, IL USA; 3 Department of Pediatrics, University of Texas Southwestern Medical Center, Dallas, TX USA; 4 Department of Biochemistry, University of Texas Southwestern Medical Center, Dallas, TX USA; 5 Cancer Center, Rush University Medical Center, Chicago, IL USA; 6 Department of Microbial Pathogens and Immunity, Rush University Medical Center, Chicago, IL USA

**Keywords:** Inflammasome, Cellular microbiology, Bacterial immune evasion, Bacterial secretion

## Abstract

Type 3 Secretion System (T3SS) is a highly conserved virulence structure that plays an essential role in the pathogenesis of many Gram-negative pathogenic bacteria, including *Pseudomonas aeruginosa*. Exotoxin T (ExoT) is the only T3SS effector protein that is expressed in all T3SS-expressing *P. aeruginosa* strains. Here we show that T3SS recognition leads to a rapid phosphorylation cascade involving Abl / PKCδ / NLRC4, which results in NLRC4 inflammasome activation, culminating in inflammatory responses that limit *P. aeruginosa* infection in wounds. We further show that ExoT functions as the main anti-inflammatory agent for *P. aeruginosa* in that it blocks the phosphorylation cascade through Abl / PKCδ / NLRC4 by targeting CrkII, which we further demonstrate to be important for Abl transactivation and NLRC4 inflammasome activation in response to T3SS and *P. aeruginosa* infection.

## Abstract (web summary)

*Pseudomonas aeruginosa* secretes the toxin ExoT, which is important for pathogenesis. Here, the authors show that ExoT inhibits NLRC4-dependent inflammatory responses during wound infection.

## Introduction

*Pseudomonas aeruginosa* is one of the most virulent opportunistic bacterial pathogens, responsible for many acute and chronic-infections in wounds, the urinary tract, burns, lungs, the bloodstream, and cystic fibrosis patients, with fatality rates reaching as high as 40%^1,2^. *P. aeruginosa* derives its versatility to cause widespread infections from arsenal of cell-associated and secreted virulence factors^3–7^. Type-3 secretion-system (T3SS) virulence apparatus arguably plays the most important role in *P. aeruginosa* pathogenesis in human^7,8^. This highly conserved injectosome enables *P. aeruginosa* to directly translocate several effector-virulence proteins into the target-host cytoplasm where they modify host cellular processes and advance *P. aeruginosa* pathogenesis^8–14^. *P. aeruginosa* strains are generally grouped as either “cytotoxic” (expressing ExoU and ExoT), or “invasive” (expressing ExoS, ExoT, and ExoY)^15,16^. Without the T3SS, both cytotoxic and invasive *P. aeruginosa* strains become severely attenuated in their ability to cause infection^8,17^, highlighting the importance of T3SS and its effector proteins in *P. aeruginosa* pathogenesis. T3SS-expressing ExoT is the only T3SS effector maintained in all T3SS-expressing *P. aeruginosa*^15^, but what makes this virulence factor indispensable to *P. aeruginosa* pathogenesis remains unclear.

Prior studies have implicated both caspase-1-dependent canonical and caspase-11-dependent noncanonical inflammasomes in the recognition of and in response to *P. aeruginosa* infection, although caspase-11 inflammasome appears to be involved only in response to *P. aeruginosa* strains lacking the T3SS^18–22^. Moreover, various inflammasome subtypes (e.g., NLRP3 and/or NLRC4) have been implicated in the recognition of and in responses to T3SS and *P. aeruginosa* infection^19,23–28^. There also appears to be some contradictory reports regarding the impact of T3SS-triggered inflammatory responses on the outcome of infection, in that the same inflammasome (NLRC4) has been shown to be either crucial in combating infection and for *P. aeruginosa* clearance, thus benefiting the host, or paradoxically enhancing *P. aeruginosa* pathogenesis and facilitating bacterial colonization, thus benefiting the pathogen^18,25,27,29–31^. Further adding to the confusion in the field, ExoS and ExoU have been shown to either dampen pro-inflammatory responses or trigger pro-inflammatory responses^25,32–38^. These reports suggest that specific organs/sites within a host may have evolved distinct mechanism(s) to detect and respond to T3SS and its effectors during *P. aeruginosa* infection. Of note, the impact of ExoT on inflammatory responses within the host has not been directly evaluated.

Wound is a favorite niche for *P. aeruginosa* infections^39–42^. *P. aeruginosa* is the number-one Gram-negative pathogen detected in both chronic and acute wounds, and its presence in wounds correlates with a poor prognosis for healing in human^43–46^. In line with these reports, *P. aeruginosa* has been shown to employ various virulence strategies to alter cellular functions and to inhibit wound healing as a way to expand its favorite niche^47–52^. Of note, T3SS plays a pivotal role in inhibiting tissue repair and exacerbating wound damage^47,48^, highlighting the importance of the T3SS function to *P. aeruginosa* pathogenesis in wounds.

In this report, we examined the impact of *P. aeruginosa* on host immune responses within the wound environment, with the focus on the T3SS and ExoT, and determined which interactions may benefit the host and which may benefit *P. aeruginosa* in this evolutionary tug of war. Our data demonstrate that recognition of *P. aeruginosa* T3SS triggers a phosphorylation cascade through the Abl/PKCδ/NLRC4, culminating in NLRC4 inflammasome activation and inflammatory responses that limit *P. aeruginosa* infection in wounds. We further show that *P. aeruginosa* ExoT dampens the phosphorylation cascade through Abl/PKCδ/NLRC4 and inhibits NLRC4 inflammasome activation by targeting CrkII, which we further demonstrate to be important for Abl transactivation and NLRC4 inflammasome activation in response to T3SS and *P. aeruginosa* infection.

## Results

### ExoT dampens pro-inflammatory cytokine production in wound

We used PA103—(a representative cytotoxic strain^7,8,53^), and PAK—(a representative invasive strain^7,8^), and their isogenic ExoT, ExoS, and ExoU T3SS mutant strains in a full-thickness excisional wound in C57BL/6 mice, as described previously^47,54^. Wounds were infected with 10^3^ wild-type or T3SS mutant strains and the impact of infection with wild-type and T3SS mutants on the production of IL-1β and IL-18 pro-inflammatory chemokines (products of inflammasomes and important pro-inflammatory cytokines against *P. aeruginosa* infection^55,56^) was assessed by enzyme-linked immunosorbent assay (ELISA) 24 h after infection.

**Fig. 1:**
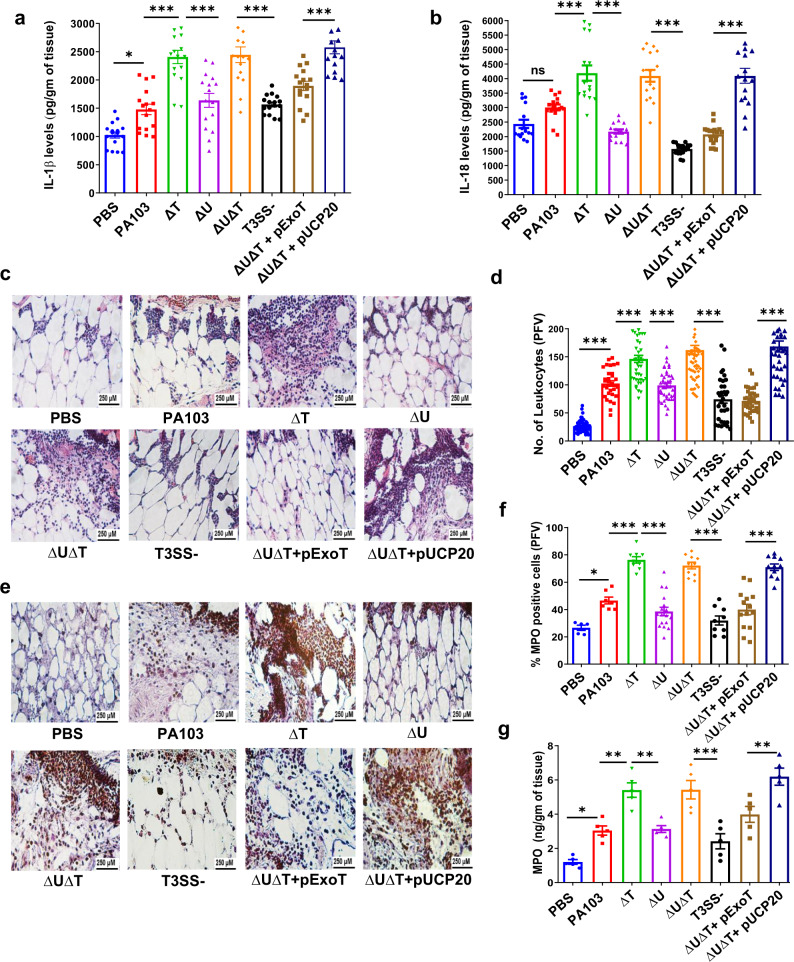
ExoT dampens inflammatory responses in wound. **a**–**g** C57BL/6 wound tissues were harvested 24 h after treatment with PBS or infection with 10^3^ wildtype *P. aeruginosa* PA103 and the indicated T3SS isogenic mutants. **a**, **b** About 24 h after infection, tissue homogenates were assessed for the pro-inflammatory cytokines IL-1β (**a**) or IL-18 (**b**) by ELISA, and the tabulated data are shown as the mean ± SEM. (*N* = 8 mice/group; 2 wounds/mouse). **c**, **d** Wounds were fixed and assessed for leukocyte migration by H&E staining. Representative images in the dermal regions from underneath the wounds are shown in (**c**) and the corresponding tabulated number of leukocytes is shown as the mean ± SEM in (**d**) (*N* = 5 mice/group; ~7 random fields/wound/mouse). **e**, **f** Neutrophil activation was assessed by determining MPO positive cells by immunohistochemistry assay (IHC). Representative images in the dermal regions from underneath the wounds are shown in (**e**) and the corresponding tabulated number of MPO-positive neutrophils is shown as the mean ± SEM in (**f**) (*N* = 5 mice/group, ~7 random fields/wound/mouse). **g** Tissue homogenates from day 1 wounds were assessed for MPO levels by ELISA (*N* = 5 mice/group). Statistical analyses were determined by one-way ANOVA with post hoc testing. ns, not significant; *p < 0.05; **p < 0.01; ***p < 0.001; ****p < 0.0001. Exact *P*-values are presented in Supplementary Data 1. Source data are provided as a Source Data file.

Infection with PA103 or PAK wild-type strains resulted in significantly more IL-1β in wounds as compared with PBS-treated uninfected wounds (Fig. 1a, Supplementary Fig. 1a, c, & e).

IL-18 was also increased in PAK-infected wounds, but not significantly increased in PA103-infected wounds (Fig. 1b & Supplementary Fig. 1b & d). Intriguingly, regardless of the strain genetic background, wounds infected with ExoT-deleted (∆T) strains, contained substantially more IL-1β and IL-18 as compared with their isogenic wildtype strains, and complementing these ∆T strains with a plasmid expressing ExoT (pExoT) reduced IL-1β and IL-18 levels significantly, suggesting that ExoT dampens inflammatory responses in wound, at least with respect to IL-1β and IL-18 inflammatory cytokines (Fig. 1a–b and Supplementary Fig. 1a–e). In contrast, wounds infected with ExoS-deleted (∆S) or ExoU-deleted (∆U) strains contained similar levels of IL-1β and IL-18 as compared with their wild-type counterparts, indicating that in wound, ExoS and ExoU do not possess anti- or pro-inflammatory functions.

### ExoT dampens leukocyte migration and activation in wound

We next assessed leukocyte infiltration in wounds treated with PBS or infected with the aforementioned *P. aeruginosa* strains, by hematoxylin and eosin (H&E) histological analysis, which is an established method for this purpose^47,54,57–60^. Consistent with the IL-1β and IL-18 chemokine levels (Fig. 1a–b and Supplementary Fig. 1a–e), wounds infected with ExoT-deleted mutant strains also contained significantly more leukocytes as compared with wild-type strains or ExoT-complemented strains (Fig. 1c–d, and Supplementary Fig. 2a–b). Corroborating these data, assessment of activated neutrophil contents in the aforementioned wounds by histological analysis of myeloperoxidase (MPO)-positive cells or by MPO measurements by ELISA also indicated significantly higher levels of activated neutrophils in wounds that were infected with ExoT-deleted mutant strains as compared with wild-type or ExoT-complemented strains (Fig. 1e–g and Supplementary Fig. 2c–d). (MPO is primarily a marker for activated neutrophils^54,58,61^).

### NLRC4 is the primary inflammasome responsible for *P. aeruginosa* recognition and it is inhibited by ExoT

**Fig. 2:**
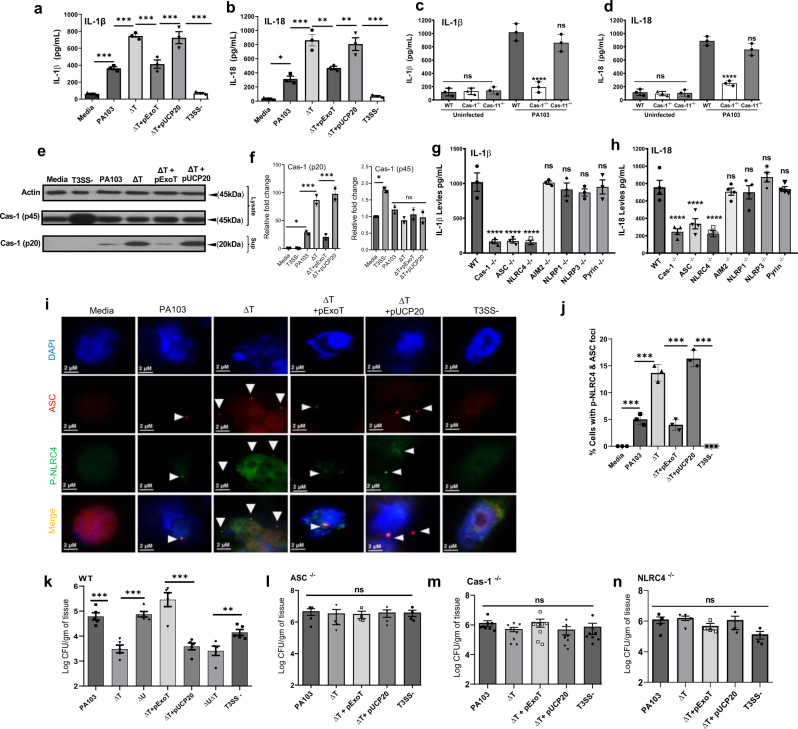
NLRC4 is the primary inflammasome responsible for *P. aeruginosa* recognition and it is inhibited by ExoT. **a**–**j** BMDMs (from C57BL/6) were infected with wild-type *P. aeruginosa* PA103 and the indicated T3SS isogenic mutants for 1-2 h (1 h for IF microscopy and 2 h for ELISA and Western blotting). **a**, **b** Culture supernatants were assessed for IL-1β or IL-18 by ELISA and the tabulated data are shown as the mean ± SEM. (*N* = 3; ns, not significant; *p < 0.05; **p < 0.01; ***p < 0.001; ****p < 0.0001). **c**, **d** BMDMs of caspase-1 or Caspase-11-knockout mice (Cas-1^−/−^ or Cas-11^−/−^) were infected with PA103. Culture supernatants were assessed for IL-1β or IL-18 by ELISA and the tabulated data are shown as the mean ± SEM. (*N* = 4; ns, not significant; *p < 0.05; **p < 0.01; ***p < 0.001; ****p < 0.0001). **e**, **f** Cell lysates and supernatants were assessed for caspase-1 activation by Western blotting (**e**) and the corresponding densitometer data are shown as the mean ± SEM in (**f**) (*N* = 3, each experiment in e & f was repeated independently 2 times. ns, not significant; *p < 0.05; **p < 0.01; ***p < 0.001; ****p < 0.0001). (**g**, **h**) BMDMs of indicated inflammasome knockout mice were infected with PA103. Culture supernatants were assessed for IL-1β or IL-18 by ELISA and the tabulated data are shown as the mean ± SEM (*N* = 4; ns, not significant; *p < 0.05; **p < 0.01; ***p < 0.001; ****p < 0.0001). (**i–j**) BMDMs (from C57BL/6) were infected with PA103 and the indicated T3SS isogenic mutants. They were then fixed and stained for *p*-NLRC4 (green), ASC (red), and nucleus/DAPI (blue). Colocalized p-NLRC4/ASC foci were assessed by IF microscopy. Representative images are shown in (**i**), and the tabulated data are shown as the mean ± SEM in (**j**) (*N* = 3 replicates, ≥7 random fields per replicate. ns, not significant; *p* < 0.05; **p < 0.01; ***p < 0.001; ****p < 0.0001). Arrows point to representative p-NLRC4/ASC foci within BMDMs. **k**–**n** Wounds of C57BL/6, ASC, Caspase-1 or NLRC4 knockout mice were infected with 10^3^ of PA103 and the indicated T3SS isogenic mutants. Bacterial burden in wounds were determined by serial dilution and plating, 24 h after infection and the tabulated data are shown as the mean ± SEM. (*N* = 5 mice/group for C57BL/6, *N* = 4 mice/group for ASC and NLRC4 knockout mice and *N* = 8 mice/group for Caspase-1 knockout mice. Statistical analyses were determined by one-way ANOVA with post hoc testing; ns, not significant; *p < 0.05; **p < 0.01; ***p < 0.001; ****p < 0.0001). Exact *P* values are presented in Supplementary Data 1. Source data are provided as a Source Data file.

ExoS and ExoU have been previously reported to dampen IL-1β production^25,32^, but the impact of ExoT on inflammasome activity in bone marrow-derived macrophages (BMDMs) has not been directly examined. Since our in vivo data clearly demonstrated that ExoT (not ExoS or ExoU) is the T3SS effector-virulence factor that dampens the production of IL-1β and IL-18 in wound (Fig. 1, Supplementary Fig. 1a–e, & Supplementary Fig. 2), we wished to assess the impact of ExoT on inflammasome activity in BMDMs. Toward this goal, we extracted BMDMs from C57BL/6 normal mice and infected them with wild-type or ExoT-deleted *P. aeruginosa* strains for 2 h. In line with our in vivo results and regardless of strain genetic background, infection with ∆T mutant strains resulted in significantly higher levels of IL-1β and IL-18 secreted by BMDMs, and complementing ∆T strains with pExoT reduced the production of these pro-inflammatory cytokines (Fig. 2a–b and Supplementary Fig. 3a–b).

Although, most studies point to the caspase-1-dependent canonical inflammasomes in mounting inflammatory responses to *P. aeruginosa* infection^18,19^, caspase-11 dependent noncanonical inflammasome has also been implicated in inflammatory responses to *P. aeruginosa*^20–22,62–64^, prompting us to reassess the role of canonical and noncanonical inflammasomes in the recognition of and mounting inflammatory responses to *P. aeruginosa* infection. Toward this goal, we challenged BMDMs extracted from C57BL/6 (WT), or caspase-1 or caspase-11 knockout mice (*Cas-1*^*–/–*^ and *Cas-11*^*–/–*^, respectively), with the *P. aeruginosa*. Culture supernatants were then examined for the production of IL-1β and IL-18 by ELISA. Data indicated that while BMDMs from WT or *Cas-11*^*–/–*^ mice recognized and responded *to P. aeruginosa* infection, *Cas-1*^*–/–*^ BMDMs were impaired in responding to *P. aeruginosa* infection, as manifested by significant reductions in IL-1β and IL-18 production (Fig. 2c–d), suggesting that *P. aeruginosa* recognition in BMDMs is primarily mediated by caspase-1 dependent canonical inflammasome. In line with ExoT’s anti-inflammatory function in wound, infection with ExoT-deleted *P. aeruginosa* strains also resulted in substantial increases in caspase-1 activation as assessed by Western immunoblotting probing for the processed active caspase-1 fragment (p20) in BMDMs, and caspase-1 inflammasome assembly as determined by caspase-1/ASC foci number determination by immunofluorescent (IF) microscopy^65^ (Fig. 2e–f and Supplementary Fig. 3c–d).

NLRP1b, NLRP3, NLRC4, AIM2, and Pyrin are the five well- characterized subtypes of caspase-1-dependent canonical inflammasomes^66,67^. Depending on the host and/or cell line, *P. aeruginosa* has been shown to activate NLRC4 or NLRP3 inflammasomes^25–28,68^, suggesting that eukaryotic hosts may utilize different caspase-1 inflammasome subtypes to mount inflammatory responses to *P. aeruginosa* infection. To determine which caspase-1-dependent canonical inflammasome subtype(s) contributed to *P. aeruginosa* recognition and response in BMDMs, we extracted BMDMs from knockout mice defective in these inflammasomes (*Nlrp1*^*–/–*^, *Nlrp3*^*–/–*^, *Nlrc4*^*–/–*^, *Aim2*^*–/–*^, and *Pyrin*^*–/–*^) and infected them with *P. aeruginosa* and assessed their supernatants for the production of IL-1β and IL-18 by ELISA. Data indicated that NLRC4 was the response in BMDMs, as defect in this inflammasome reduced IL-1β and IL-18 production to levels comparable to *Cas-1*^*–/–*^ BMDMs (Fig. 2g–h and Supplementary Fig. 3e). Moreover, infection with ExoT-deleted mutant strains resulted in nearly 3-fold increase in the number of NLRC4 inflammasome foci and complementing this strain with pExoT, but not empty vector, significantly reduced NLRC4 inflammasome foci numbers in BMDMs (Fig. 2i–j), indicating that ExoT interferes with NLRC4 inflammasome assembly.

### ExoT enhances bacterial fitness in the wounds by dampening the NLRC4 inflammasome

We next determined bacteria (infection) burden in the wounds infected with the wildtype or T3SS mutant strains by colony-forming unit (CFU) determination, as described^47,54,69,70^. Data indicated that regardless of genetic background, ExoT-deleted mutant strains were significantly impaired in their ability to colonize wound, as manifested by approximately 1–2 log-order reductions in bacterial counts in wound as compared with the wild-type strains (Fig. 2k and Supplementary Fig. 3f). Complementing ∆T mutant strains with pExoT, (but not with the empty vector pUCP20), restored their ability to colonize the wound, suggesting that heightened inflammatory responses in wounds are responsible for the reduction in the fitness of ExoT-deleted mutant strains in this environment. Of note, ExoS-deleted mutants also exhibited reduced colonization in wounds, but ExoU-deleted strains did not, indicating that ExoS (but not ExoU), imparts additional fitness benefit to *P. aeruginosa* in wound. In line with this notion, PAK strain consistently colonized wounds ~1 log order more than PA103 strain (Supplementary Fig. 3h), suggesting that PAK’s enhanced colonization in wound may be due to its possession of ExoS virulence factor.

To assess the role of caspase-1-dependent NLRC4 inflammasome function in controlling *P. aeruginosa* infection in wound, we challenged wounds in mice deficient in caspase-1 (*Cas-1*^*–/–*^), or the adaptor protein ASC (*ASC*^*–/–*^), which is required for all caspase-1 dependent canonical inflammasome subtypes^71^, or NLRC4 (*Nlrc4*^*–/–*^), with the aforementioned *P. aeruginosa* strains. Regardless of strain genetic background, wounds in *Cas-1*^*–/–*^, *ASC*^*–/–*^, and *Nlrc4*^*–/–*^ mice were significantly more vulnerable to infection with *P. aeruginosa* as they contained >1 log-order more bacteria than wounds in C57BL/6 normal mice (Fig. 2k–n, and Supplementary Fig. 3f–g). Moreover, the presence or absence of T3SS or ExoT did not impact the ability of *P. aeruginosa* to colonize wounds in caspase-1- or NLRC4 inflammasome defective mice, indicating that the primary virulence function of ExoT in wound is to dampen inflammatory responses through NLRC4 inflammasome (Fig. 2k–n, and Supplementary Fig. 3f–g).

*P. aeruginosa* infection in C57BL/6 wounds peaks on day 3, but begins to resolve by day 6^47^. We conducted a similar time-course infection study in normal and NLRC4^−/−^ mice to assess the role of NLRC4 inflammasome in combating *P. aeruginosa* infection. Data indicated that NLRC4^−/−^ wounds contained significantly more bacteria than normal wounds on days 1, 3, and 6, highlighting the importance of NLRC4 in combating *P. aeruginosa* infection in wound (Supplementary Fig. 3i). Collectively, these data indicated that T3SS-expressing *P. aeruginosa* recognition in wound by NLRC4 canonical inflammasome results in pro-inflammatory responses that limit *P. aeruginosa* infection. The data further indicated that ExoT functions as the main anti-inflammatory agent for *P. aeruginosa*, providing a necessary stealth mechanism against the host innate immune responses for this pathogen in wound environment.

### NLRC4 phosphorylation by PKCδ plays a critical role for NLRC4 inflammasome activation in response to *P. aeruginosa* infection

**Fig. 3:**
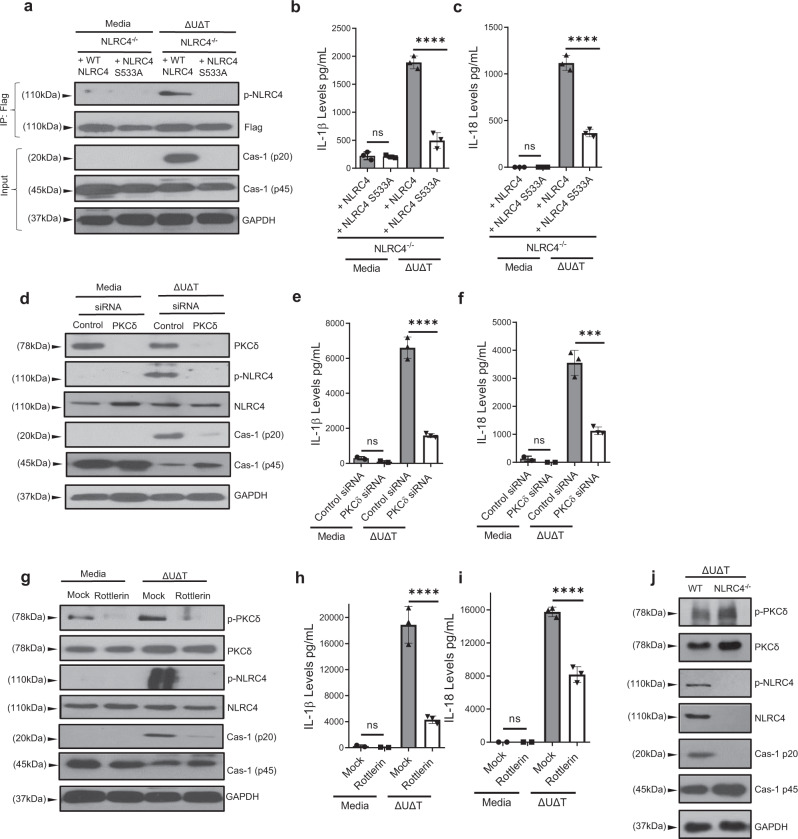
NLRC4 phosphorylation and PKCδ play critical roles for NLRC4 inflammasome activation in response to *P. aeruginosa* infection. **a–c** *Nlrc4*^−/−^ BMDMs were reconstituted with wildtype NLRC4 or nonphosphorylatable NLRC4 mutant (NLRC4/S533A). They were then infected with *P. aeruginosa* ΔUΔT (T3SS^+^) strain for 2 h. Cell lysates and supernatants were assessed for caspase-1 activation by Western blotting (**a**) and culture supernatants were assessed for IL-1β or IL-18 by ELISA and the tabulated data are shown as the Mean ± SEM in (**b**, **c**) respectively. **d–f** BMDMs from C57BL/6 were transfected for PKCδ siRNA for 48 h. After transfection, cells were infected with *P. aeruginosa* ΔUΔT for 2 h. Cell lysates were assessed for indicated proteins (phosphorylated and unphosphorylated forms) by Western blotting (**d**) and culture supernatants were assessed for IL-1β or IL-18 cytokines by ELISA, and the tabulated data are shown as the mean ± SEM in (**e**, **f**) respectively. **g**–**i** BMDMs from C57BL/6 were pretreated with PKCδ inhibitor Rottlerin (5 µM) for 90 min and then cells were infected with *P. aeruginosa* ΔUΔT for 2 h. Whole-cell lysate was assessed for indicated proteins (phosphorylated and unphosphorylated forms) by Western blotting (g) and culture supernatants were assessed for IL-1β and IL-18 cytokines by ELISA and the tabulated data are shown as the mean ± SEM (**h**, **i**), respectively. **j** BMDMs (from C57BL/6 and *Nlrc4*^−^^/−^) were infected with ΔUΔT for 2 h. Cell lysates were assessed for indicated proteins (phosphorylated and unphosphorylated forms) by Western blotting. (Statistical analyses were determined by one-way ANOVA with post hoc testing. Each experiment was repeated independently three times, except (**a**), which was repeated twice (*N* = 3 each time). ns, not significant; * *p* < 0.05; **p < 0.01; ***p < 0.001; ****p < 0.0001). Exact *P*-values are presented in Supplementary Data 1. Source data are provided as a Source Data file.

There appears to be some controversy regarding the role of NLRC4 phosphorylation at serine residue 533 (Ser 533) in NLRC4 inflammasome activity, with some groups reporting it to be essential for NLRC4 inflammasome activation^72–74^, while others questioning the dependence of NLRC4 inflammasome activity on the NLRC4 phosphorylation^75,76^. To evaluate the dependence of NLRC4 inflammasome activation on NLRC4 phosphorylation in response to *P. aeruginosa* infection, we reconstituted NLRC4^−/−^ BMDMs with either wild-type NLRC4 or nonphosphorylatable NLRC4 mutant (NLRC4/S533A)^72^ (Methods), and assessed their responses to *P. aeruginosa* infection. Data indicated that NLRC4/S533A-expressing BMDMs were significantly impaired in responding to *P. aeruginosa* infection as assessed by Western blotting, probing for activated caspase-1 (p20), and IL-1β and IL-18 production (Fig. 3a–c, and Supplementary Fig. 4a). These data indicated that NLRC4 phosphorylation is required for NLRC4 inflammasome activation in response to *P. aeruginosa* infection.

NLRC4 phosphorylation at Ser 533 is mediated by PKCδ kinase and has been shown to be essential for NLRC4 inflammasome activity in BMDMs infected with *Salmonella enterica* serovar Typhimurium (a.k.a., *Salmonella* Typhimurium)^72^. However, the role of PKCδ in NLRC4 inflammasome activation has also been questioned^75,76^, prompting us to assess the dependence of NLRC4 inflammasome activity on PKCδ kinase activity in response to *P. aeruginosa*. PKCδ protein depletion by treatment with PKCδ-specific siRNA^77^, or pretreatment of BMDMs with PKCδ kinase inhibitor Rottlerin^72^, significantly reduced NLRC4 phosphorylation, caspase-1 activation, and IL-1β and IL-18 production, in BMDMs infected with *P. aeruginosa* (Fig. 3d–i and Supplementary Fig. 4b–c). Of note, while caspase-1 processing/activation was substantially reduced in NLRC4^−^^/−^ BMDMs infected with *P. aeruginosa*, PKCδ phosphorylation was not affected, indicating that PKCδ is upstream of NLRC4 (Fig. 3j and Supplementary Fig. 4d). These results indicated that PKCδ kinase activity is essential for NLRC4 phosphorylation and NLRC4 inflammasome activation in response to *P. aeruginosa*.

### NLRC4 inflammasome inhibition by ExoT is primarily dependent on its ADPRT-domain activity

**Fig. 4:**
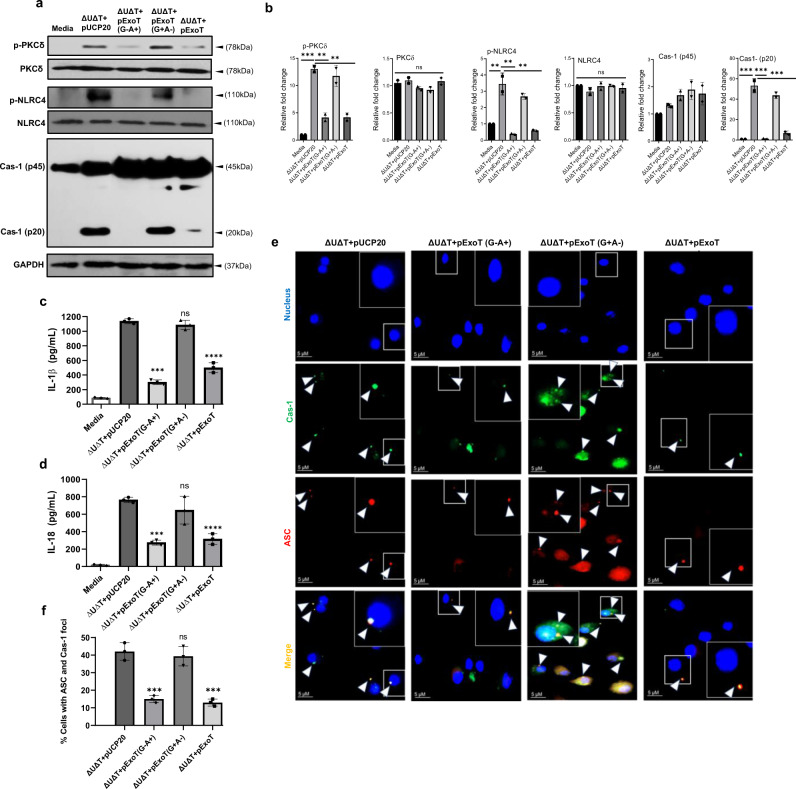
NLRC4 inflammasome inhibition by ExoT is dependent on its ADPRT domain activity. **a**, **b** BMDMs from C57BL/6 were infected with indicated *P. aeruginosa* strains for 2 h. Supernatants and cell lysates were then assessed for indicated phosphorylated and unphosphorylated proteins by Western blotting (**a**), and the corresponding densitometer data are shown in (**b**) as the mean ± SD. (Each experiment in a–b was repeated independently at least two times) (*N* = 3, ns, not significant; *p < 0.05; **p < 0.01; ***p < 0.001; ****p < 0.0001). **c**, **d** IL-1β and Il-18 cytokines were assessed by ELISA and the tabulated data are shown as the mean ± SD. (Each experiment was done twice independently) (*N* = 3 replicates each time). ns, not significant; *p* < 0.05; **p < 0.01; ***p < 0.001; ****p < 0.0001. **e**, **f** Colocalized cas*p*ase-1/ASC foci were assessed by IF microsco*p*y. Representative images are shown in (**e**) and the tabulated data are shown as the mean ± SD in (**f**). Arrows point to some caspase-1/ASC foci within BMDMs. Insets are the magnified images of representative caspase-1/ASC foci. The scale bars are 5 µm. (*N* = 3 replicates; ≥7 random fields per replicate. ns, not significant; *p < 0.05; **p < 0.01; ***p < 0.001; ****p < 0.0001. Statistical analyses were determined by one-way ANOVA with post hoc testing). Exact *P-*values are *p*resented in Supplementary Data 1. Source data are provided as a Source Data file.

ExoT is a bifunctional virulence factor that contains a GTPase-activating protein (GAP) domain at its N-terminus and an ADP-ribosyltransferase (ADPRT) domain at its C-terminus^78,79^. To gain insight into the mechanism by which ExoT interferes with NLRC4 inflammasome activity, we assessed the contributions of the GAP and the ADPRT domains in mediating ExoT’s anti-inflammasome function in BMDMs. Data indicated that ExoT—in a manner that was primarily dependent on its ADPRT-domain activity—dampened PKCδ and NLRC4 phosphorylation, inhibited caspase-1 processing, interfered with IL-1β and IL-18 production, and interfered with caspase-1 inflammasome assembly (Fig. 4a–f). In line with these results, ExoT’s anti-inflammatory function in wound was also mediated by its ADPRT-domain activity, as mutation in the ADPRT domain activity abrogated ExoT’s ability to dampen IL-1β production or enhance *P. aeruginosa* colonization in wound (Supplementary Fig. 5a–b). However, the underlying mechanism responsible for inhibition of NLRC4 inflammasome by ExoT/ADPRT domain remained unclear.

### ExoT/ADPRT blocks NLRC4 inflammasome by dampening the phosphorylation cascade through NLRC4 inflammasome by targeting Crk adaptor protein

PKCδ itself becomes phosphorylated at tyrosine residue 311 (Tyr 311) and PKCδ phosphorylation has been shown to be required for NLRC4 phosphorylation and NLRC4 inflammasome activation in BMDMs in response to infection with *Salmonella* Typhimurium^72^, but the identity of the tyrosine kinase responsible for PKCδ phosphorylation has remained unknown for nearly a decade. ExoT/ADPRT domain has been demonstrated to ADP-ribosylate and inactivate Crk adaptor protein^9,10,52,79^. Interestingly, CrkI and CrkII isoforms of Crk adaptor protein were originally identified by their homology to the oncogene product of avian sarcoma virus CT10 (v-Crk)—which despite the absence of an apparent catalytic domain in the oncogene—induces massive increases in tyrosine-phosphorylated proteins in chicken-embryo fibroblasts transformed by this virus^80,81^. We posited that ExoT may inhibit *P. aeruginosa*-triggered NLRC4 activation by targeting Crk adaptor protein that we further postulated to be essential (either directly or indirectly) for PKCδ phosphorylation and for NLRC4 inflammasome activation.

**Fig. 5:**
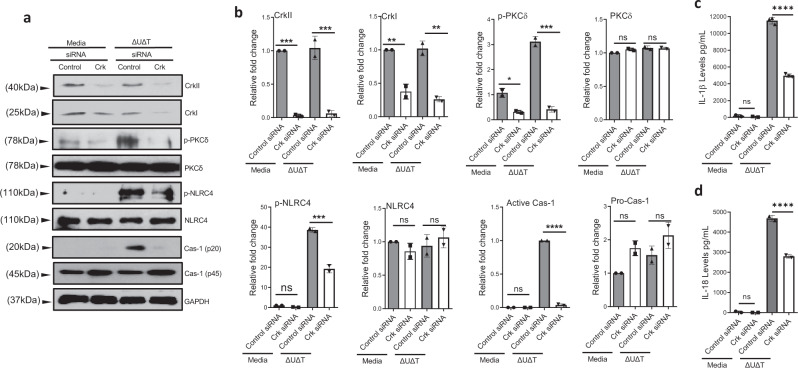
Crk adaptor protein is required for NLRC4 inflammasome activation. **a–d** BMDMs from C57BL/6 were transfected for Crk siRNA for 48 h. After transfection, cells were infected with *P. aeruginosa* ΔUΔT strain for 2 h. Whole-cell lysate was assessed for indicated proteins (phosphorylated and unphosphorylated forms) by Western blotting (**a**), and the corresponding densitometer data are shown as the mean ± SEM in (**b**). (Each experiment in a, b was repeated at least three times independently). **c**, **d** Culture supernatants were assessed for IL-1β and IL-18 cytokines by ELISA and the tabulated data are shown as the Mean ± SEM. (Each experiment was repeated at least 3 times independently with *N* = 3 replicates each time; ns, not significant; *p < 0.05; **p < 0.01; ***p < 0.001; ****p < 0.0001). Statistical analyses were determined by one-way ANOVA with post hoc testing. Exact *P*-values are presented in Supplementary Data 1. Source data are provided as a Source Data file.

To test our hypothesis, we first evaluated the dependence of PKCδ and NLRC4 phosphorylation and NLRC4 inflammasome activation on Crk in response to *P. aeruginosa*, by depleting BMDMs of Crk adaptor protein by Crk-specific siRNA^9,82^ prior to infection with *P. aeruginosa*. Treatment with Crk siRNA reduced the levels of CrkI and CrkII isoforms by >90%, and significantly reduced PKCδ and NLRC4 phosphorylation, and caspase-1 processing as assessed by Western blotting (Fig. 5a–b), and significantly dampened NLRC4 inflammasome activity as assessed by IL-1β and IL-18 analysis by ELISA (Fig. 5c–d). These data indicated that Crk adaptor protein plays a significant role in PKCδ and NLRC4 phosphorylation and for NLRC4 inflammasome activation in response to *P. aeruginosa* infection, but how Crk mediated NLRC4 inflammasome activation in response to *P. aeruginosa* infection remained unclear.

### Crk-dependent Abl tyrosine-kinase activity is required for PKCδ phosphorylation and NLRC4 inflammasome activation in response to *P. aeruginosa* infection

**Fig. 6:**
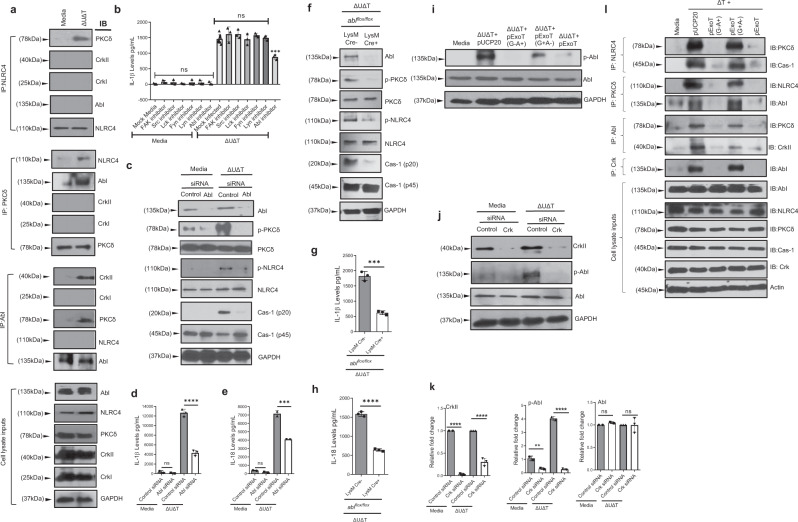
Crk-induced Abl tyrosine-kinase activity is required for PKCδ phosphorylation and NLRC4 inflammasome activation in response to *P. aeruginosa* infection. **a** BMDMs from C57BL/6 were infected with the indicated *P. aeruginosa* strain for 2 h. Whole-cell lysates were immunoprecipitated with antibodies against NLRC4, PKCδ or Abl and immunoblotted for indicated proteins. **b** BMDMs from C57BL/6 were pretreated with inhibitors of indicated Crk-interacting tyrosine kinases (Methods) for 90 min and then infected with ΔUΔT for 2 h, and the supernatants were assessed for IL-1β and IL-18 by ELISA and the tabulated data are shown as the mean ± SEM. **c–e** BMDMs from C57BL/6 were transfected with Abl siRNA for 24 h. After transfection, cells were either left uninfected (media) or infected with the indicated *P. aeruginosa* strain for 2 h. Whole-cell lysates were assessed for indicated proteins (phosphorylated and unphosphorylated forms) by Western blotting (**c**), and culture supernatants were assessed for IL-1β and IL-18 by ELISA, and the tabulated data are shown as the mean ± SEM in (**d**, **e**), respectively. **f–h** BMDMs from *Abl*^flox/flox^ *LysM Cre-* and *Abl*^flox/flox^ *LysM Cre* + mice were infected with the indicated *P. aeruginosa* strain for 2 h. Whole-cell lysates were assessed for indicated proteins (phosphorylated and unphosphorylated forms) by Western blotting (**f**), and culture supernatants were assessed for IL-1β and IL-18 cytokines by ELISA and the tabulated data are shown as the mean ± SEM in (**g**, **h**), respectively. **i** BMDMs from C57BL/6 were infected with indicated *P. aeruginosa* strains for 2 h. Whole cell lysates were assessed for indicated proteins (phosphorylated and unphosphorylated forms) by Western blotting. **j**, **k** BMDMs from C57BL/6 were transfected with Crk siRNA for 48 h. After transfection, cells were infected with indicated *P. aeruginosa* strain for 2 h. Whole cell lysate was assessed for indicated proteins (phosphorylated and unphosphorylated forms) by Western blotting (j) and the corresponding densitometer data are shown as the mean ± SEM in (**k**). **l** BMDMs from C57BL/6 were infected with indicated *P. aeruginosa* strains for 2 h. Whole-cell lysates and supernatants were immunoprecipitated with antibodies against NLRC4, PKCδ, Crk or Abl and immunoblotted for indicated proteins. (Experiments were repeated twice independently, except (**c**–**e**), which were repeated at least 3 times independently with *N* = 3 replicates each time. ns, not significant; *p < 0.05; **p < 0.01; ***p < 0.001; ****p < 0.0001. Statistical analyses were determined by one-way ANOVA with post hoc testing for all except (**g**, **h**), which were done by two-sided unpaired Student’s *t-*test). Exact *P*-values are presented in Supplementary Data 1. Source data are provided as a Source Data file.

To hone in on the role of Crk in NLRC4 inflammasome activity, we next evaluated the interaction between Crk and PKCδ and NLRC4 by co-immunoprecipitation (Co-IP)-pulldown assay, using PKCδ or NLRC4 as baits. While PKCδ and NLRC4 co-immunoprecipitated with each other in response to infection as expected, neither protein co-immunoprecipitated with CrkI or CrkII isoforms of Crk (Fig. 6a and Supplementary Fig. 6a), suggesting that Crk functions upstream of PKCδ phosphorylation. FAK, Src, Lck, Fyn, Lyn, and Abl are all tyrosine kinases, which have been shown to either directly or indirectly interact with Crk under various conditions^83–88^. We wondered whether one of these tyrosine kinases may be responsible for PKCδ phosphorylation and NLRC4 activation in response to *P. aeruginosa* infection. To test our hypothesis, we infected BMDMs with *P. aeruginosa* after pretreating them with specific inhibitors of the aforementioned tyrosine kinases^89–95^. Data indicated that pretreatment with Abl inhibitor (Imatinib) significantly reduced IL-1β production in BMDMs infected with ExoT-deficient *P. aeruginosa* (Fig. 6b). Corroborating these data and ruling out off-target effects of Imatinib, Abl-depleted BMDMs, after treatment with Abl-specific siRNA (^96^ and Methods), and Abl-knockout BMDMs, extracted from *Abl*^*flox/flox*^ *LysM Cre* + mice (^97^ and Methods), also displayed significant reductions in PKCδ and NLRC4 phosphorylation, caspase-1 activation, and IL-1β and IL-18 production in BMDMs infected with *P. aeruginosa* (Fig. 6c–h, and Supplementary Fig. 6b–c).

Intriguingly, Abl co-immunoprecipitated with PKCδ but not with NLRC4 in response to infection with *P. aeruginosa* (Fig. 6a and Supplementary Fig. 6a). Moreover, Abl phosphorylation in response to *P. aeruginosa* infection was not affected in BMDMs pretreated with PKCδ inhibitor Rottlerin^72^ or *Nlrc4*^*–/–*^ or in *Cas-1*^*–/–*^ BMDMs, while PKCδ inhibition abrogated NLRC4 phosphorylation and caspase-1 activation in response to *P. aeruginosa* infection (Supplementary Fig. 7a–d, Fig. 3d–j, and Supplementary Fig. 4b–c). These data further support the notion of Abl functioning upstream of PKCδ, mediating its phosphorylation, and PKCδ functioning upstream of NLRC4, mediating its phosphorylation in response to infection with *P. aeruginosa*.

Abl-kinase interaction with Crk adaptor protein has been shown to result in Abl activation by autophosphorylation (transactivation) at tyrosine 245 (Tyr 245)^98,99^. Intriguingly, Abl also co-immunoprecipitated with CrkII isoform of Crk in response to *P. aeruginosa* infection (Fig. 6a and Supplementary Fig. 6), suggesting that Abl interaction with CrkII may be important for Abl transactivation in BMDMs in response to *P. aeruginosa* infection. To address this possibility, we first assessed whether Abl becomes phosphorylated at Tyr 245 in BMDMs in response to *P. aeruginosa*. Infection with *P. aeruginosa* strain resulted in substantial increases in Abl phosphorylation at Tyr 245, which was significantly inhibited by ExoT/ADPRT (Fig. 6i and Supplementary Fig. 6d). Consistent with Crk’s role in Abl transactivation^98,99^, Crk-protein depletion by siRNA led to ~90% reduction in Abl phosphorylation in BMDMs infected with *P. aeruginosa* (Fig. 6j–k). These data indicated that Abl transactivation in response to *P. aeruginosa* infection is dependent on its interaction with Crk adaptor protein, and suggested that Crk/Abl interaction is disrupted by ExoT/ADPRT.

To assess the interactions between ExoT with Crk and/or Abl, we transfected BMDMs with a mammalian expression vector, harboring ExoT or inactive mutant ExoT (ExoT G^−^A^−^), fused to GFP at their C-termini, in the presence of pancaspase inhibitor Z-VAD to block ExoT/GAP and ExoT/ADPRT-induced apoptosis^10,100,101^. (These vectors have been described previously and GFP fusion does not alter ExoT activities^9,10,52,100,102^). Data demonstrated that ExoT-GFP and ExoT(G^−^A^−^)–GFP both interacted with CrkI and CrkII isoforms of Crk but not with Abl, as assessed by Co-IP, using GFP as the bait (Supplementary Fig. 8a–b), indicating that ExoT does not directly interact with Abl. Given that CrkII only interacted with Abl in response to infection (Fig. 6a), we next infected the aforementioned transfected BMDMs with ∆U∆T *P. aeruginosa* strain and assessed the interaction between ExoT–GFP and ExoT(G^−^A^−^)–GFP with Crk and Abl in the aforementioned transfected BMDMs under infection. ExoT or ExoT(G^−^A^−^) co-immunoprecipitated with both CrkI and CrkII in response to infection (Supplementary Fig. 8c–d). In contrast, Abl co-immunoprecipitated with ExoT(G^−^A^−^) but significantly less with ExoT, suggesting that Abl interaction with ExoT (G^−^A^−^) is likely indirect through CrkII and ExoT interferes with CrkII/Abl interaction (Supplementary Fig. 8c–d).

To further explore this possibility, we next assessed the impact of ExoT on CrkII/Abl interaction by Co-IP pull-down assay in BMDMs infected with ExoT-deleted or ExoT-expressing strains. Data indicated that ExoT disrupted CrkII/Abl interaction in a manner that was primarily dependent on its ADPRT-domain activity (Fig. 6l and Supplementary Fig. 9). These data are consistent with ExoT’s inhibition of Abl transactivation that is dependent on its interaction with CrkII (Fig. 6i–k). Of note, interactions between Abl/PKCδ and PKCδ/NLRC4 were also significantly disrupted by ExoT in a manner that was dependent on its ADPRT activity, indicating that these interactions are dependent on CrkII/Abl interaction and Abl transactivation (Fig. 6l and Supplementary Fig. 9).

We next assessed the involvement of Abl and PKCδ in caspase-1 inflammasome activity toward WI-14 and WI-16 *P. aeruginosa* wound clinical isolates, which are respectively similar to PA103 and PAK strains in regard to their T3SS effectors^15^. Infection with these clinical strains also led to caspase-1 activation in BMDMs and significant increases in IL-1β and IL-18 production (Supplementary Fig. 10a–d). Moreover, Abl and PKCδ protein depletion by their specific siRNAs also significantly dampened IL-1β and IL-18 production in BMDMs infected with these clinical isolates (Supplementary Fig. 10e–g), further highlighting the importance of Abl and PKCδ in inflammatory responses to T3SS-expressing *P. aeruginosa*.

### Abl kinase is required for NLRC4 inflammasome activation in response to *P. aeruginosa* infection in wound

**Fig. 7:**
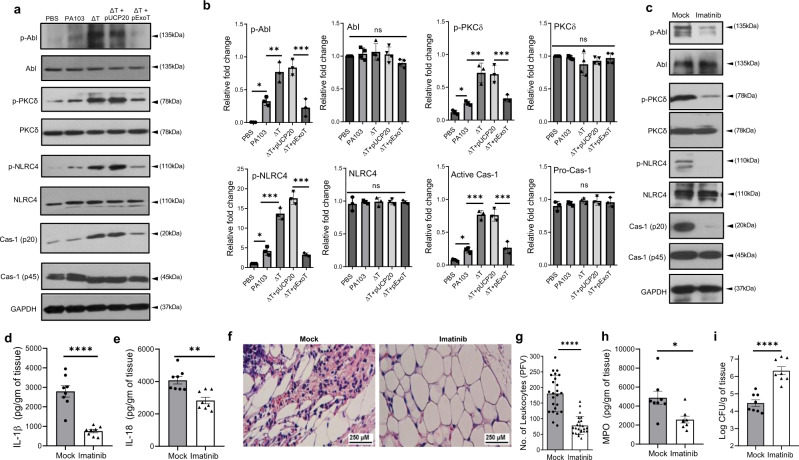
Abl is essential for *P. aeruginosa* recognition and infection control in wound, and it is inhibited by ExoT. **a**, **b** C57BL/6 wound tissues were harvested 24 h after treatment with PBS or infection with 10^3^ WT PA103, and ExoT-deleted (∆T) isogenic strains with or without ExoT complementation. Tissue homogenates were assessed for indicated proteins (phosphorylated and unphosphorylated forms) by Western blotting and the corresponding densitometer data are shown as the mean ± SEM (*N* = 3). Statistical analyses were determined by one-way ANOVA with post hoc testing, ns, not significant; *p < 0.05; **p < 0.01; ***p < 0.001; ****p < 0.0001. **c–i** C57Bl/6 mice received Imatinib (100 mg/kg) or PBS (mock) by i.p injection (Materials). Wounds were infected with 10^3^ of *P. aeruginosa* ∆U∆T strain. After 24 h of infection, tissue homogenates were assessed for indicated proteins by Western blotting (**c**) and for the pro-inflammatory cytokines by ELISA, and the tabulated data are shown as the mean ± SEM (**d**, **e**) (*N* = 8). (**f**, **g**) Wounds were fixed and assessed for leukocyte migration by H&E staining. Representative images in the dermal regions from underneath the wounds are shown in (**f**) and the corresponding tabulated number of leukocytes is shown as the mean ± SEM in (**g**) (*N* = 5 mice/group, ~7 random fields/wound/mouse). **h** Tissue homogenates from day-1 wounds were assessed for MPO levels by ELISA. **i** Bacterial burden was determined by serial dilution and plating after 24 h of infection and the tabulated data are shown as the mean ± SEM (*N* = 8; ns, not significant; *p < 0.05; **p < 0.01; ***p < 0.001; ****p < 0.0001. Statistical analyses were determined by two-sided unpaired Student *t*-test). Exact *P*-values are presented in Supplementary Data 1. Source data are provided as a Source Data file.

To assess the importance of Abl kinase function in NLRC4 inflammasome activation in response to *P. aeruginosa* infection in wound, we first assessed the phosphorylation status of Abl, PKCδ, and NLRC4 in wound infected with ExoT-deleted or ExoT-expressing *P. aeruginosa* strains. Data demonstrated that wounds infected with wild-type *P. aeruginosa* contained significantly higher levels of phosphorylated Abl, PKCδ, and NLRC4, and activated caspase-1 (p20) as compared with uninfected wounds (Fig. 7a–b), indicating that NLRC4 inflammasome becomes activated in wound in response to *P. aeruginosa* infection. Consistent with the in vitro data in BMDMs, wounds infected with ExoT-deleted mutants contained significantly higher levels of phosphorylated Abl, PKCδ, and NLRC4, and activated caspase-1 (p20), as compared with wounds infected with wild-type *P. aeruginosa* strain or ExoT-deleted strain complemented with ExoT (Fig. 7a–b), indicating that ExoT dampens NLRC4 inflammasome activation in wound.

Abl deletion causes lethality at embryonic day 8–9 because of Abl’s crucial roles in vascular function and in development^103,104^. To assess the role of Abl kinase in NLRC4 inflammasome activity in response to *P. aeruginosa* infection in wound, we treated C57BL/6 mice with Abl inhibitor Imatinib or PBS by intraperitoneal injection, prior to wounding and infection with *P. aeruginosa* (Methods). We next assessed the wounds for inflammatory responses and infection burden 24 h after infection. As expected, wounds in Imatinib-treated mice contained significantly lower levels of phosphorylated Abl (Fig. 7c and Supplementary Fig. 11). Consistent with the role of Abl kinase in PKCδ phosphorylation and NLRC4 inflammasome activation in response to *P. aeruginosa* (Fig. 6b–h and Supplementary Fig. 6a–c), Imatinib-treated wounds also contained significantly lower levels of phosphorylated PKCδ and NLRC4, and activated caspase-1 (p20) as assessed by Western blotting (Fig. 7c and Supplementary Fig. 11); reduced IL-1β and IL-18, as assessed by ELISA (Fig. 7d–e); reduced leukocyte content as assessed by H&E staining (Fig. 7f–g); and reduced activated neutrophils as assessed by MPO analysis by ELISA (Fig. 7h). Importantly, Imatinib-treated mice contained nearly 2 log-order more bacteria in their wounds, as compared with mock-treated mice (Fig. 7i). Collectively, these data indicated that Abl function is essential for NLRC4 inflammasome activation and in combating *P. aeruginosa* infection in wound.

## Discussion

In this report, we assessed the impact of *P. aeruginosa* infection on inflammatory responses within the wound environment. Our data show that recognition of T3SS-expressing *P. aeruginosa* strains by the caspase-1 dependent canonical inflammasome leads to pro-inflammatory responses, manifested by increases in caspase-1-activation, IL-1β, and IL-18 cytokine production, and leukocyte migration to the site of infection. Our data further indicate that NLRC4 canonical inflammasome subtype is primarily responsible for the recognition of and response to *P. aeruginosa* infection in BMDMs and in wound. Interestingly, NLRC4 inflammasome has been shown to either protect the host against *P. aeruginosa* infection or paradoxically facilitate and enhance *P. aeruginosa* colonization and pathogenesis^18,31^. Our data indicate that in wound, NLRC4 inflammasome activity provides crucial protection against *P. aeruginosa* infection, as wounds in mice defective in the components of NLRC4 inflammasome are highly vulnerable to *P. aeruginosa* infection.

There appears to be some controversy regarding the role of PKCδ kinase and NLRC4 phosphorylation in NLRC4 inflammasome activity, with some groups reporting them to be essential^72–74^, while others disputing their role in NLRC4 inflammasome activity^75,76^. These seeming differences may reflect the different microorganisms used in these studies (e.g., *Salmonella, Shigella, and Legionella*); the different experimental procedures used for analyses (e.g., BMDM infection with bacteria, vs. delivering bacterial products, such as flagellin by FlaTox, vs. in vitro reconstitution assays); and the complexity of inflammasome responses emerging when multiple factors (e.g., T3SS, T4SS, or flagellin) are analyzed independently or in combination, as have been the case in these studies. Our data clearly demonstrate that NLRC4 phosphorylation by PKCδ is essential for NLRC4 activation and pro-inflammatory responses to *P. aeruginosa* infection. We show that NLRC4 is phosphorylated in response to *P. aeruginosa*, both in vitro and in vivo. We further show that BMDMs expressing nonphosphorylatable NLRC4 are impaired in their ability to respond to *P. aeruginosa*. We also demonstrate that PKCδ-depleted BMDMs and BMDMs treated with PKCδ kinase inhibitor Rottlerin fail to phosphorylate NLRC4 and activate NLRC4 inflammasome in response to *P. aeruginosa* infection.

PKCδ phosphorylation has also been shown to occur and be required for the NLRC4 inflammasome activity in BMDMs in response to T3SS of *S*. Typhimurium^72^. However, the identity of the kinase-mediating PKCδ phosphorylation has remained unknown for nearly a decade. We show that during infection with *P. aeruginosa* and in response to T3SS, Abl tyrosine kinase interacts with PKCδ and this interaction is required for PKCδ phosphorylation and for NLRC4 inflammasome activation. Abl has not been implicated in inflammasome activity or in tissue defenses against *P. aeruginosa*. Moreover, we show that during infection with *P. aeruginosa* and in response to T3SS, CrkII isoform of Crk adaptor protein interacts with Abl tyrosine kinase and this interaction is required for Abl kinase autophosphorylation (transactivation) and for the subsequent phosphorylation cascade through Abl → PKCδ → NLRC4, and ultimately for NLRC4 inflammasome assembly and activity. Crk adaptor protein has not been implicated in inflammasome activity or pro-inflammatory responses to *P. aeruginosa*. Of note, while Abl co-immunoprecipitated with both CrkII and PKCδ and PKCδ co-immunoprecipitated with Abl and NLRC4 in response to *P. aeruginosa* T3SS, CrkII and PKCδ or Abl and NLRC4 did not co-immunoprecipitate with each other, suggesting that these interactions are either transient or they are mutually exclusive as they may involve the same interaction domains in these proteins. More studies are needed to tease out the nature of these interactions.

ExoT is the only T3SS effector protein that is expressed in all T3SS-expressing *P. aeruginosa* clinical isolates^15,105^, suggesting a more fundamental role for this virulence factor in *P. aeruginosa* pathogenesis. In this report, we demonstrate that ExoT is the main anti-inflammatory agent for *P. aeruginosa* in wound and without ExoT, *P. aeruginosa* is substantially attenuated in its ability to colonize wound, due to heightened inflammatory responses triggered by caspase-1-dependent NLRC4 inflammasome. ExoT has not been implicated in inflammasome regulation or inflammatory responses and ExoT’s anti-inflammatory activity is perhaps the most important function for this virulence factor, adding to its growing virulence activities that make this virulence factor indispensable to *P. aeruginosa* pathogenesis^9,10,12,52,100,102^. Interestingly, we also find ExoS (but not ExoU), to contribute to *P. aeruginosa* fitness in wound. ExoU has been shown to significantly enhance *P. aeruginosa* fitness in systemic infection^25^. Combined, these data suggest that ExoS and ExoU enhance *P. aeruginosa*’s capabilities to expand its niches within the host during infection.

Imatinib treatment in cancer patients suffering from chronic myelogenous leukemia (CML), gastrointestinal stromal tumors (GISTs), and other types of cancers^106,107^, has been linked to increased bacterial infection, such as *P. aeruginosa*^108–112^, suggesting that increased vulnerability to infection in these patient cohorts could be due to dampened NLRC4 inflammasome activity.

**Fig. 8:**
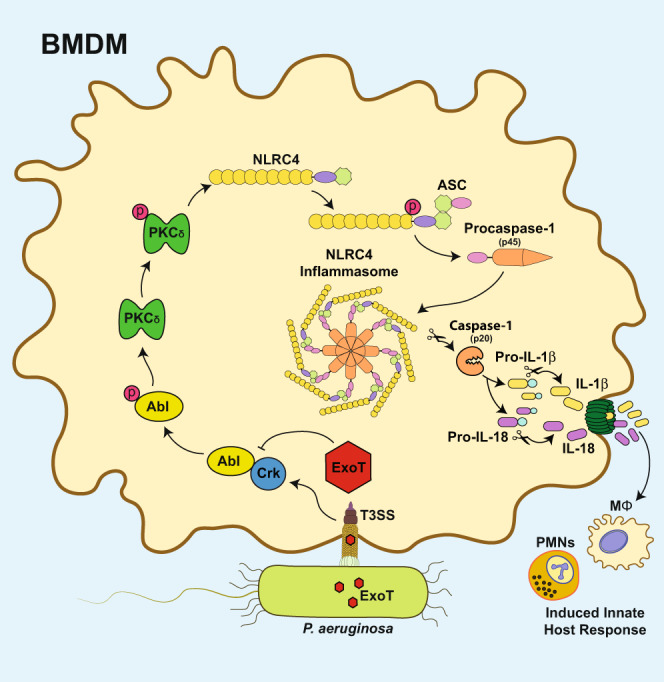
ExoT dampens inflammatory responses by blocking phosphorylation cascade through NLRC4 inflammasome (model). Insertion of T3SS in target BMDMs results in CrkII/Abl interaction which induces Abl transactivation/autophosphorylation. Activated Abl kinase then activates PKCδ kinase by phosphorylation, which in turn activates NLRC4 by phosphorylation, which then in association with ASC and pro-caspase-1 forms NLRC4 inflammasome, culminating in caspase-1 activation by proteolysis. Activated caspase-1 then processes pro-IL-1β and pro-IL-18 into mature IL-1β and IL-18, which are then secreted and in turn initiate inflammatory responses by recruiting leukocytes, such as neutrophils (PMNs) and macrophages (Møs) to the site of infection where they combat infection. *P. aeruginosa* ExoT dampens NLRC4 inflammasome by interfering with CrkII/Abl interaction and by blocking the phosphorylation cascade through Abl→PKCδ→NLRC4 needed for NLRC4 assembly and function.

In summary (Fig. 8), we report that *P. aeruginosa* T3SS triggers a rapid phosphorylation cascade involving CrkII/Abl → PKCδ → NLRC4, which leads to caspase-1 activation, culminating in the production of IL-1β and IL-18 pro-inflammatory cytokines, which in turn mobilize inflammatory leukocytes, such as neutrophils (PMNs) and macrophages (Møs), to the site of infection where they combat *P. aeruginosa* infection. *P. aeruginosa* solves this dilemma by deploying ExoT, which inhibits NLRC4 inflammasome activation by disrupting CrkII/Abl interaction, which in turn interferes with the phosphorylation cascade through the Abl/PKCδ/NLRC4 inflammasome. By functioning as an anti-inflammatory agent, ExoT enables *P. aeruginosa* to keep its T3SS and add other T3SS effector-virulence factors (i.e., ExoS and ExoU), which further enhance its ability to expand its niches within the host.

## Methods

### Procedures related to animal studies

Our research complies with all relevant ethical regulations from Rush Institutional Animal Care and Use Committee. We have an approval from Rush Institutional Animal Care and Use Committee (IACUC No: 18-013) to conduct research as indicated. All procedures complied strictly with the standards for care and use of animal subjects as stated in the Guide for the Care and Use of Laboratory Animals (Institute of Laboratory Animal Resources, National Academy of Sciences, Bethesda, MD, USA). Mice were allowed to acclimate to the environment for 1 week prior to experimentation. Mice were maintained in a 12-/12-h dark/light cycle at controlled temperature (22 °C ± 1.5) and humidity conditions (40–60%) and were fed standard *ad libitum* diet (Teklad 2018). About 7-week-old C57BL/6 (000664), *Nlrp1*^*–/–*^ (021301), *Nlrp3*^*–/–*^ (021302), *Cas-1*^*–/–*^ (032662), *Cas-11*^*–/–*^ (024698), *Pyrin*^*+/–*^ (*Mefv*^*+/–*^) (021315), and *Aim2*^*–/–*^ (013144) mice were obtained from the Jackson Laboratories (Bar Harbor, ME). *Pyrin*^*+/–*^ (*Mefv*^*+/–*^) mice were intercrossed to generate *Pyrin*^*–/–*^ (*Mefv*^*–/–*^)-knockout mice. *ASC*^*–/–*^ and *Nlrc4*^*–/–*^ knockout mice were kind gifts from Dr. Vishva Dixit (Genentech, San Francisco, CA). All knockout mice were confirmed by genotyping. *Abl* ^flox/flox^ *LysM Cre-* and *Abl* ^flox/flox^ *LysM Cre* + were generated by crossing mice carrying floxed Abl alleles with mice carrying the LysM Cre allele^97,113^. All mice were backcrossed to C57BL/6 at least 6 times during generation. Femur and tibia bones of *Abl* ^flox/flox^ *LysM Cre-* and *Abl* ^flox/flox^ *LysM Cre* + were harvested as described^111^. (For detailed information on mice and their sources see Supplementary Table 1).

### Bacteria preparation

(For a complete list of bacteria, plasmids, and their sources used in these studies, refer to Supplementary Table 1). Strains were either in PA103 or PAK genetic backgrounds and were previously described (^10,13,100,114^ & in Supplementary Table 1). WI-14 and WI-16 wound clinical *P. aeruginosa* isolates were generous gifts from Dr. Alan Hauser (Northwestern University) and were described previously^15^. *P. aeruginosa* strains were cultured in Luria–Bertani broth overnight, diluted 1:40 (volume/volume), and grown for 2 h. Bacteria were washed and resuspended in PBS.

### Wounding and wound infection

Wounding was carried out as described previously^47,54,57,115^. A sterile biopsy punch (5-mm diameter) was used to punch through the full thickness of the back skin below the shoulder blades of mice. Bacteria were prepared as described above and 1000 bacteria (resuspended in 10 μL of saline solution) were added to each wound. Mice were housed in individual cages after wounding to prevent cross-contamination. In an experiment evaluating the role of Abl kinase in vivo, C57BL/6 mice were injected interperitoneally (i.p) with 100 mg/kg Imatinib or saline at 48, 24, and 2 h before wounding and bacterial infection. Bacteria numbers in wounds were determined as described previously^47,54,69,70^. Briefly, wound tissues were harvested at indicated timepoints after wounding and infection. Tissues were cut into small pieces with sterile scissors and digested with collagenase D for 40 min at 37 °C. The total number of bacteria per gram of wound tissue was then determined by serial dilution and plating on LB agar plates after normalization to tissue weight.

### Bone marrow-derived macrophages (BMDMs)

BMDMs were extracted from femurs and tibias of indicated mice as described^57,115^. Cells were seeded in 96- or 6-well plates. Cells were primed with ultrapure LPS (Invivogen, USA) for 2–6 h and infected with *P. aeruginosa* strains at indicated multiplicity of infection (M.O.I) of 10–20 for 1–2 h as indicated in the figure legends. The lower M.O.I and the lower duration of infection was used for the studies involving NLRC4/ASC/caspase-1 inflammasome assessments by IF microscopy, as ExoU is highly toxic to cells^116^. In experiments involving the assessments of tyrosine kinases’ role in NLRC4 inflammasome, BMDMs were pretreated with indicated inhibitors: FAK inhibitor, 1,2,4,5-benzenetetraamine tetrahydrochloride at 100 μM concentration^89^; Src inhibitor, PP2 at 10 μM concentration^90,91^; Lck inhibitor, TC-S7003 at 10 μM concentration^92^; Fyn inhibitor, AZD0530 at 10 μM concentration^93^; Lyn inhibitor, Lyn peptide inhibitor at 20 μM concentration^94^; Abl inhibitor, Imatinib at 30 μM^95^; or PKCδ inhibitor, Rottlerin at 5 µM, for 90 min before bacterial infection.

### Cytokine analyses

Tissue homogenates and cell culture supernatants were assessed for the pro-inflammatory cytokines IL-1β and IL-18 by ELISA (Invitrogen, USA) according to the manufacturer’s instructions, as we described^54,57,60^.

### Histopathological evaluation

Leukocytes’ infiltration in wounds was assessed by immunohistochemical (IHC) analyses using H&E, as described previously^47,54,57,58,117^. Neutrophil activation in wounds was assessed by IHC analysis using MPO staining (anti-Myeloperoxidase antibody) (Abcam Cat. No. ab9535, 1:200)^54,58,60,70^. The histological data were obtained from *N* ≥ 5 mice/group and ≥7 random fields/wound/mouse.

### Western blot analyses

Western blot analysis were performed as described previously^13,52,114,118^. Briefly, the supernatants of treated and/or infected BMDMs were subjected to trichloroacetic acid protein precipitation. The precipitates were denatured in Laemmli buffer and analyzed by Western blot for activated caspase-1 and IL-1β, using their respective antibodies. Cell lysates were immunoblotted for pro-caspase-1 and pro-IL-1β or immunoblotted for indicated proteins (phosphorylated and unphosphorylated forms) using their respective antibodies.

### Antibodies

Anti-caspase-1 (P20) (AdipoGen Cat. No. AG20B0042C100, 1:1000), anti-ASC pab (AL177) (AdipoGen Cat. No. AG25B0006C100, 1:1000), Crk Mouse Monoclonal Antibody Clone: 22 (BD Cat. No. 610035, 1:1000), NLRC4 (Abcam Cat. No. ab201792 and Millipore Sigma Cat. No. 06-112-5MI, 1:200), Phospho-NLRC4 (SER-533) PAB (ECM Biosciences Cat. No. NP5411, 1:50), Phospho-NLRC4 (Ser533) Mouse anti-Human, Mouse, Clone: 4B7B7 (Invitrogen Cat. No. PIMA531846, 1:50), Polyclonal Anti-PKCδ (Cell Signaling Technology Cat. No. 2058, 1:1000), Phospho-PKCδ (Tyr311) Antibody (Cell Signaling Technology Cat. No. 2055, 1:500), and Mouse IL-1β Antibody (R&D systems Cat. No. AF-401-NA, 1:1000). Mouse (MOPC-21) mAb IgG1 Isotype Control (Cell Signaling Technology, Cat. No. 4097, 1:1000) NLRC4 Polyclonal Antibody Invitrogen (Cat. No. PA5-72908, 1:100), Anti- c-Abl (24-11) (Santa Cruz Biotechnology Cat. No. sc-23, 1:100), donkey anti-goat IGG Secondary Antibody (HRP) (Novus Biologicals Cat. No. NBP1-74815, 1:1000), β-Actin (13E5) Rabbit mAb (Cell Signaling Technology, Cat. No. 4970, 1:2000) Anti-Myeloperoxidase antibody (Abcam Cat. No. ab9535, 1:1000), Anti-ABL1 (phosphor-Y245) (Abcam Cat. No. ab193223, 1:100), anti-rabbit IGG, HRP-linked Antibody (Cell Signaling Technology, Cat. No. 7074 S, 1:5000), Anti-mouse IgG, HRP-linked Antibody (Cell Signaling Technology, Cat. No. 7076, 1:5000), and GAPDH Antibody Rabbit Polyclonal (Proteintech Cat. No. 1094-I-AP, 1:5000).

### Immunoprecipitation

BMDMs were infected with indicated *P. aeruginosa* strains at M.O.I of 20 for 2 h. Whole-cell lysates were immunoprecipitated with antibodies against NLRC4 (NLRC4 Polyclonal Antibody (Invitrogen Cat. No. PA5-72908 and Abcam Cat. No. ab201792, 1:50)), PKCδ (Cell Signaling Technology, Cat. No. 2058, 1:50) and Crk (BD Cat. No. 610035, 1:50), and Abl (Santa Cruz Biotechnology Cat. No. sc-23, 1:50) according to the manufacturer’s instructions (Direct and Classic ip kits, Pierce). Immunoprecipitated lysates were immunoblotted for indicated proteins as discussed earlier.

### Protein depletion by siRNA transfection

Protein depletion by siRNA was performed as described^9,82,119^. Briefly, BMDMs were transfected with Crk siRNA, PKCδ siRNA, and Abl siRNA (final concentration, 100 nM) using a lipofectamine RNAimax reagent (Invitrogen, USA) according to the manufacturer’s instructions for 48 h, 48 h, and 24 h, respectively. After transfection, cells were washed with PBS to remove transfection regents and resuspended in fresh media for the indicated experiments. Cells were primed with ultrapure LPS (200 ng/mL) for 2 h. They were then infected with indicated *P. aeruginosa* strains at M.O.I. of 20 for 2 h. Whole-cell lysates were assessed for indicated proteins (phosphorylated and unphosphorylated forms) by Western blotting, and the corresponding supernatants were assessed for IL-1β and IL-18 cytokines by ELISA as discussed earlier.

### *Nlrc4*^*−/−*^ cells’ reconstitution

*Nlrc4*^*−/−*^ cells (obtained from Genentech) were reconstituted with wild-type NLRC4 or nonphosphorylatable NLRC4 mutant (NLRC4/S533A) vectors, as described^72^ and according to the manufacturer’s instructions (Cell Biolabs). Reconstituted cells were infected with *P. aeruginosa* (M.O.I. 20) for 2 h. Whole-cell lysates were immunoprecipitated with antibodies against Flag (Millipore Sigma, Cat. No. F1804, 1:50) and assessed for indicated proteins (phosphorylated and unphosphorylated forms) by Western blotting. The culture supernatants were subjected to trichloroacetic acid protein precipitation. The precipitates were denatured in Laemmli buffer and analyzed by Western blot for activated caspase-1. The culture supernatants were assessed for IL-1β and IL-18 cytokines by ELISA as discussed earlier.

**Transient transfection** was performed as previously described^13,52,102^. Briefly, BMDMs were pretreated with a pan-caspase inhibitor (Z-VAD) at 60 µM final concentration 1 h prior to transfection with indicated plasmids (ExoT-GFP, ExoT (G^-^A^+^)–GFP, ExoT (G^+^A^−^)–GFP, and GFP) to prevent ExoT/GAP or ExoT/ADPRT-induced apoptosis^10,100^. About 24 h after transfection, cells were either treated with PBS (uninfected) or infected with indicated *P. aeruginosa* strains for 2 h. Whole-cell lysates were immunoprecipitated with antibodies against GFP (Abcam, Cat. No. AB5450, 1:50) and immunoblotted for indicated proteins.

### Fluorescence microscopy

Immunofluorescent microscopy was performed as described^12,13,82,114^. Briefly, BMDMs were seeded in coated coverslips in 24-well plates. After LPS priming and bacterial infection, coverslips were fixed with 4% PFA for 20 mins, and permeabilized using 0.1% Triton X-100 and blocked for 60 mins at room temperature. Coverslips were stained with antibodies against ASC (AdipoGenCat. No. AG25B0006C100, 1:200), Caspase-1 (AdipoGen Cat. No. AG20B0042C100, 1:200), and p-NLRC4 (Invitrogen Cat. No. PIMA531846, 1:100) at 1:100 dilution overnight at 4 °C. After washing with 1X PBS, coverslips were then incubated with goat anti-mouse IgG (H + L) secondary antibody, Alexa Fluor 488 (Invitrogen Cat. No. A-11001, 1:500), goat anti-rabbit IgG (H + L) secondary antibody, and Texas Red (Invitrogen Cat. No. T-6391, 1:500), at 1:500 dilution for 1 h at room temperature in the dark. Coverslips were washed three times with 1 X PBS, mounted with DAPI nuclear dye, and visualized under fluorescence microscope. These studies were done in triplicates, and for each replicate, ≥7 randomly selected fields were counted, and data were presented as the mean ± SEM.

### Data collection

Imaging data were collected on Axiovert Z1 microscope (ZEISS), using ZEISS ZEN blue edition software.

### Data analyses

We used GraphPad Prism version 8 (GraphPad software), Excel (Microsoft Office, version, Professional Plus 2016), Image J (open source, version, 1.52a**)**, and Illustrator 2021 (Adobe, version, 25.4.3), to analyze the data in this study.

### Sample size

Sample size was determined based on our previous experience^12,54,57,117,120^ and was large enough to detect differences between samples with biological significance.

### Quantification and statistical analysis

Statistical analyses between two groups were performed by two-sided unpaired Student’s *t*-test and statistical analyses of more than two groups were performed by one-way analysis of variance (ANOVA) with additional post hoc testing, using the GraphPad Prism software, version 8. Data are presented as mean ± SEM or mean ± SD. *P*-values less than or equal to 0.05 were taken as significant.

### Reporting summary

Further information on research design is available in the Nature Research Reporting Summary linked to this article.

## Supplementary information

Supplementary Information

Reporting Summary

Description of Additional Supplementary Files

Supplementary Data 1

## Data Availability

A “Reporting Summary” for this article is available as Supplementary Information file. The main data supporting the findings of this study are available within the article and its Supplementary Figures. The source data underlying all figures and Supplementary Figures are provided as a Source Data file. Source data are provided with this paper.

## Source data

Source Data
