# Supplementary material for: CrkII/Abl phosphorylation cascade is critical for NLRC4 inflammasome activity and is blocked by Pseudomonas aeruginosa ExoT
Source: Nat Commun. 2022 Mar 11;13:1295. doi: 10.1038/s41467-022-28967-5 (PMC8917168; doi:10.1038/s41467-022-28967-5)
Supplement: Supplementary file 1 — Supplementary Information [file 41467_2022_28967_MOESM1_ESM.pdf]

Supplementary Information

Title

CrkII/Abl phosphorylation cascade is critical for NLRC4 inflammasome activity and is blocked by *Pseudomonas aeruginosa* ExoT

Author list

Mohamed F. Mohamed [1,2], Kajal Gupta [1,2], Josef W. Goldufsky [1,2], Ruchi Roy [1,2], Lauren T. Callaghan [5,6], Dawn M. Wetzel [5,6], Timothy M. Kuzel [1,2,4], Jochen Reiser [1], and Sasha H. Shafikhani [1,2,3,4]*

Affiliations

[1] Department of Medicine, [2] Division of Hematology/Oncology/Cell Therapy, [3] Department of Microbial Pathogens and Immunity, [4] Cancer Center, Rush University Medical Center, Chicago, IL, USA.

[5] Department of Pediatrics, [6] Department of Biochemistry, University of Texas Southwestern Medical Center, Dallas, TX, USA.

* To whom correspondence should be addressed: Sasha_Shafikhani@rush.edu

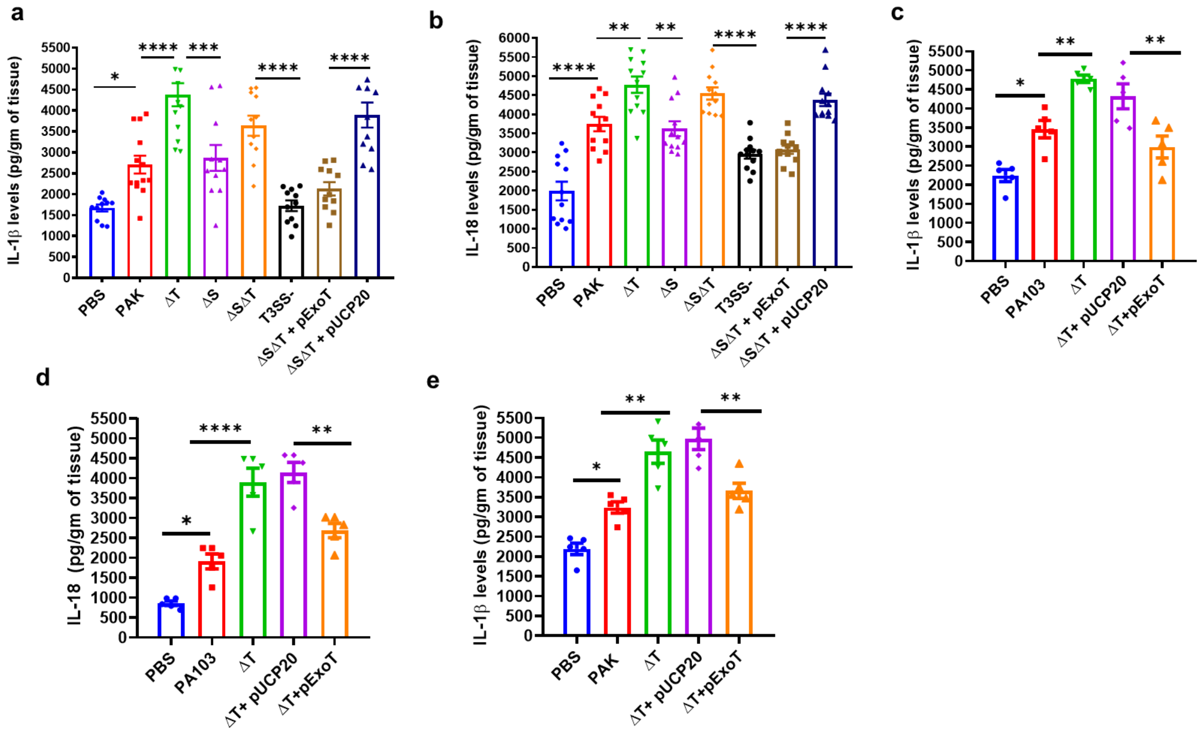

Supplementary Fig. 1. ExoT dampens pro-inflammatory cytokine production in wound. (a-e) C57BL/6 wound tissues were harvested 24h after treatment with PBS or infection with 10^3 of indicated PAK or PA103 and their T3SS isogenic mutants. Tissue homogenates were assessed for the pro-inflammatory cytokines IL-1β or IL-18 by ELISA, and the tabulated data are shown as the Mean ± SEM. (N=8 mice/group for a-b; N=5 mice/group for c-e; * $p<0.05$; ** $p<0.01$; *** $p<0.001$; **** $p<0.0001$. Statistical analyses between groups were performed by One-way analysis of variance (ANOVA) with additional post hoc testing. Exact *P* values are presented in Supplementary Data 1. Source data are provided as a Source Data file.

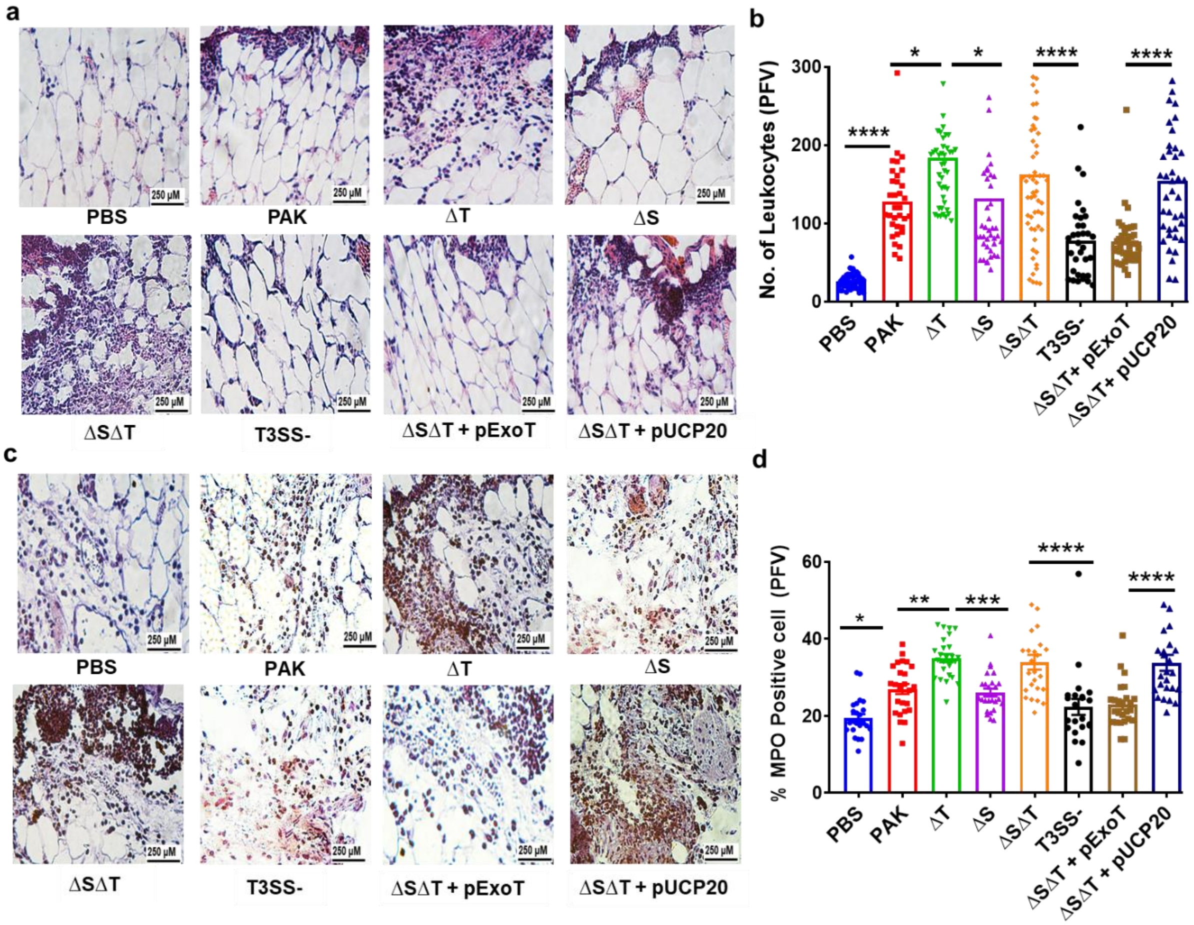

Supplementary Fig. 2. ExoT dampens leukocyte migration and activation in wound during infection. (a-d) C57BL/6 wound tissues were harvested 24h after treatment with PBS or infection with 10^3 PAK or the indicated T3SS isogenic mutants. (a-b) Wounds were fixed and assessed for their leukocyte content by H&E staining. Representative images in the dermal region from underneath the wounds are shown in (a), and the corresponding tabulated number of leukocytes is shown as the Mean ± SEM in (b) (N=5 mice/group; ~7 random fields/wound/animal; **p*<0.05; ***p*<0.01; ****p*<0.001; *****p*<0.0001). (c-d) Neutrophil influx was assessed by determining MPO positive cells by immunohistochemistry (IHC). Representative images in the dermal region from underneath the wounds are shown in (c) and the corresponding tabulated data are shown as the Mean ± SEM in (d). (N=5 mice/group; ~7 random fields/wound/animal; **p*<0.05; ***p*<0.01; ****p*<0.001; *****p*<0.0001. Statistical analyses between groups were performed by One-way ANOVA with additional post hoc testing). Exact *P* values are presented in Supplementary Data 1. Source data are provided as a Source Data file.

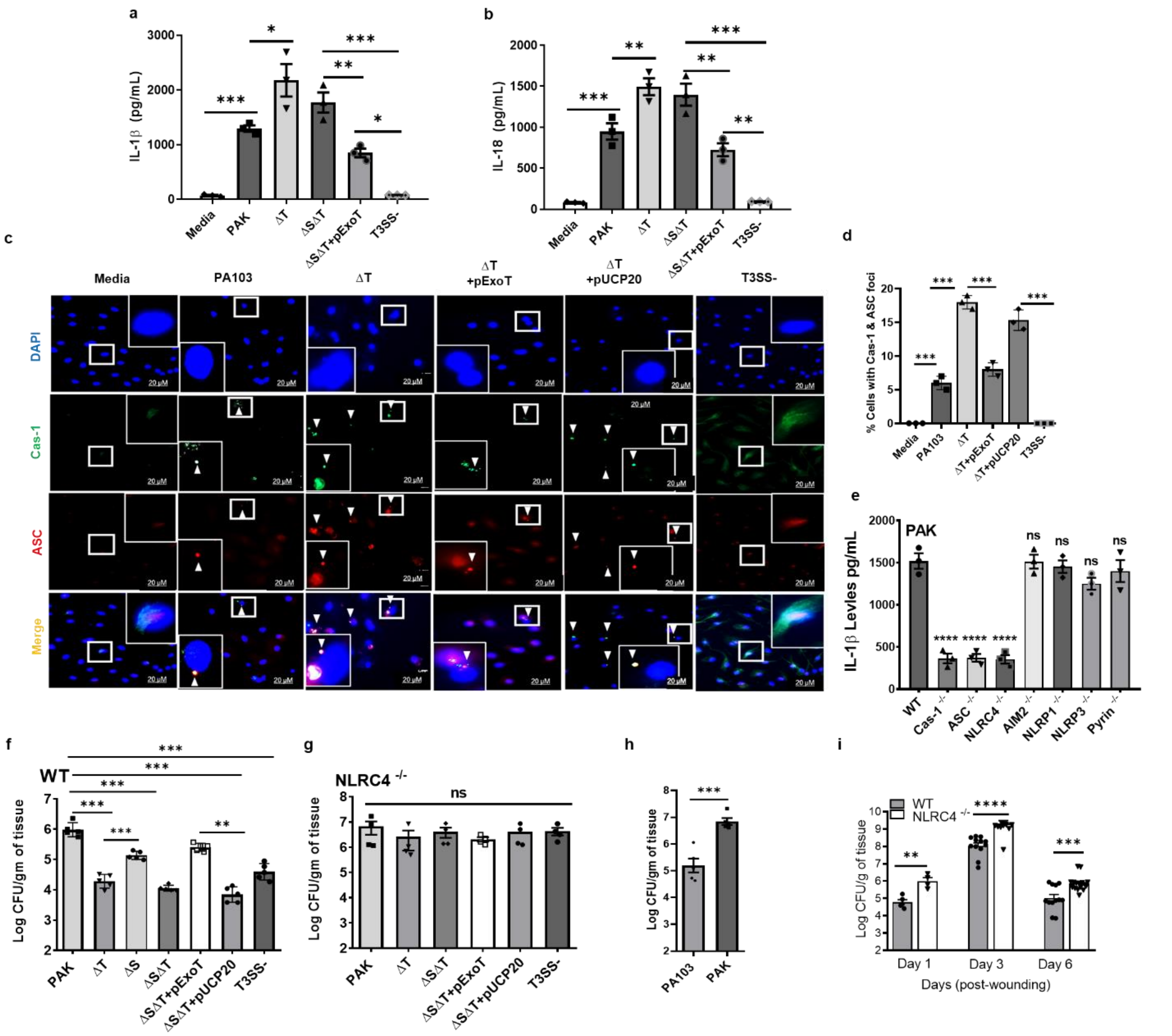

Supplementary Fig. 3. NLRC4 is the primary inflammasome responsible for *P. aeruginosa* recognition and it is inhibited by ExoT. (a-e) BMDMs (from C57BL/6) were infected with wildtype PAK and the indicated T3SS isogenic mutants for 2h. (a-b) Culture supernatants were assessed for IL-1β or IL-18 cytokine by ELISA the tabulated are shown as the Mean ± SEM in (a & B) respectively. (N=3; ns, Not Significant; p<0.05; **p<0.01; ***p<0.001; ****p<0.0001. Statistical analyses between groups were performed by One-way ANOVA with additional post hoc testing). (c-d) BMDMs (from C57BL/6) were infected with PA103 and

indicated T3SS isogenic mutants for 1h. They were then fixed and stained for Caspase-1 (green), ASC (red), and nucleus/DAPI (blue). Colocalized p-NLRC4/ASC foci were assessed by IF microscopy. Representative images are shown in (c), and the tabulated data are shown as the Mean ± SEM in (d). (N=3 replicates; ≥7 random fields per replicate. ns, Not Significant; *$p<0.05$; **$p<0.01$; ***$p<0.001$; ****$p<0.0001$). Arrows point to some Caspase-1 /ASC foci within BMDMs. e) BMDMs of indicated inflammasome knockout mice were infected with PAK. Culture supernatants were assessed for IL-1β cytokine by ELISA, 2h after infection and the tabulated data are shown as the Mean ± SEM. (N=4; ns, Not Significant; $p<0.05$; **$p<0.01$; ***$p<0.001$; ****$p<0.0001$). (f-g) Wounds of C57BL/6 and NLRC4 knockout mice were infected with 10^3 PAK and the indicated T3SS isogenic mutants. Bacterial burden in wounds were determined by serial dilution and plating, 24h after infection and the tabulated data are shown as the Mean ± SEM. (N=5 mice/group for C57BL/6 and N=4 mice/group for NLRC4 knockout; ns, Not Significant; $p<0.05$; **$p<0.01$; ***$p<0.001$; ****$p<0.0001$). (h) Wounds of C57BL/6 mice were infected with 10^3 PA103 and PAK. Bacterial burden in wounds were determined by serial dilution and plating, 24h after infection and the tabulated data are shown as the Mean ± SEM. (N=5 mice/group; $p<0.05$; **$p<0.01$). Statistical analyses were performed by two-sided unpaired Student's *t*-test. (i) Wounds of C57BL/6 and NLRC4 knockout mice were infected with 10^3 of PA103. Bacterial burden in wounds were determined by serial dilution and plating, at day 1, 3 and 6 after infection and the tabulated data are shown as the Mean ± SEM. (N=5 mice/group *$p<0.05$; **$p<0.01$; ***$p<0.001$; ****$p<0.0001$). Exact *P* values are presented in Supplementary Data 1. Source data are provided as a Source Data file.

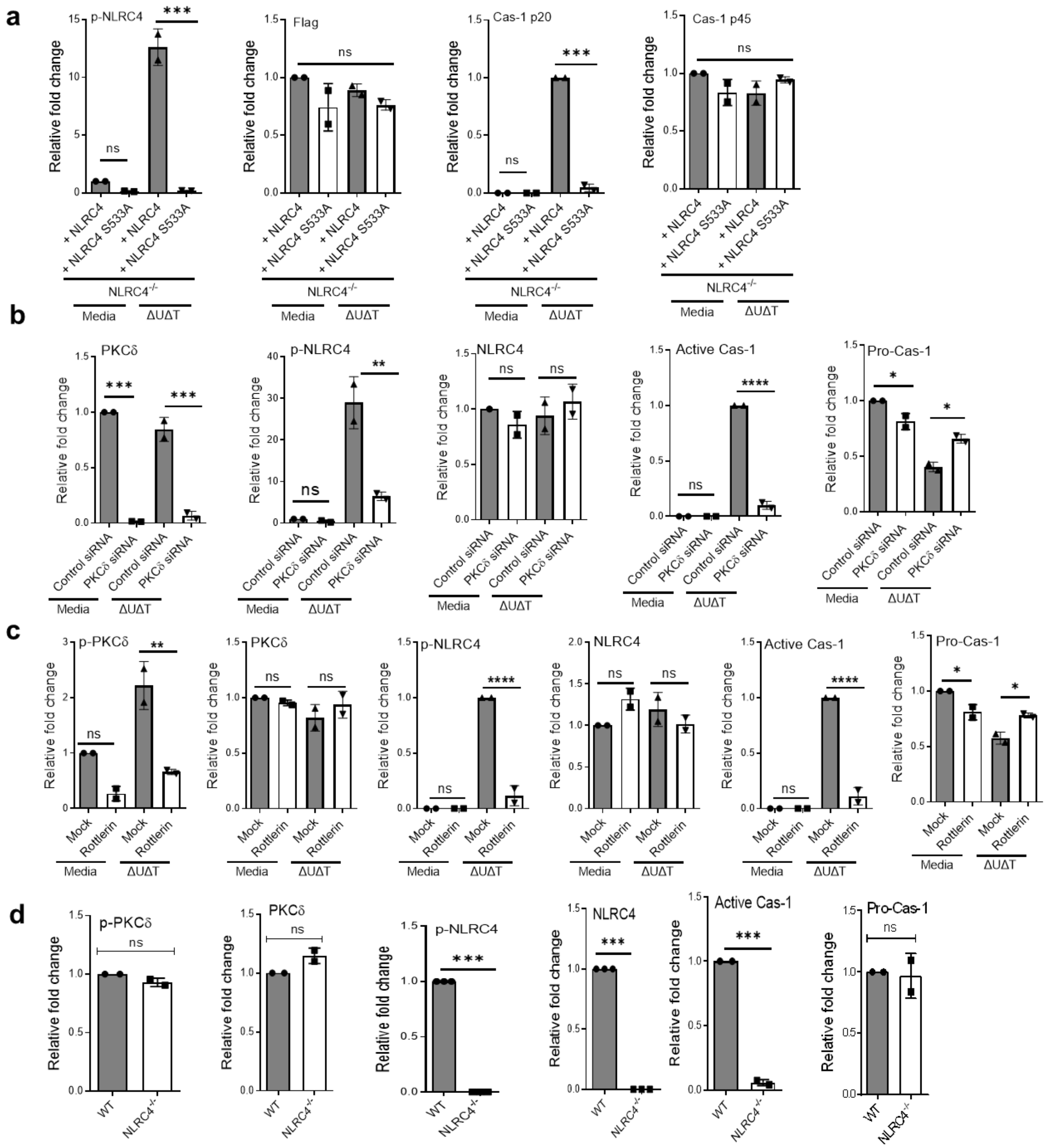

f

Supplementary Fig. 4. Densitometry values associated with Figure 3. The corresponding densitometer data associated with Fig. 3 are shown as the Mean ± SEM. (Statistical analyses between groups were performed by One-way ANOVA with additional post hoc testing, and statistical analyses in (d) were performed by two-sided unpaired Student's *t*-test. Each experiment was repeated 3 times independently, except (a) which was repeated independently

twice. ns, Not Significant; *$p<0.05$; **$p<0.01$; ***$p<0.001$; ****$p<0.0001$)**.** Exact *P* values are presented in Supplementary Data 1. Source data are provided as a Source Data file.

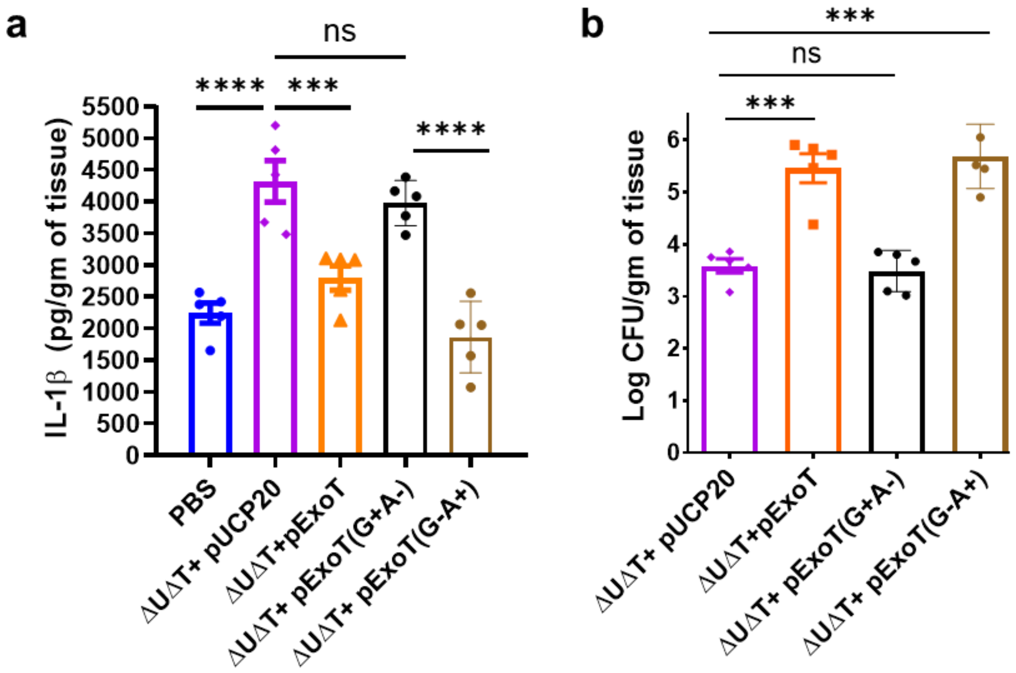

Supplementary Fig. 5. **ADPRT domain activity of ExoT is necessary for ExoT-mediated dampening of pro-inflammatory cytokines and *P. aeruginosa* survival in wound**. (a-b) C57BL/6 wound tissues were harvested 24h after treatment with PBS or infection with 10^3 of indicated *P. aeruginosa* strains. (a) Tissue homogenates were assessed for the pro-inflammatory cytokines IL-1β by ELISA, and the tabulated data are shown as the Mean ± SEM (N=5 mice/group). (b) Bacterial burden was determined by serial dilution and plating and the tabulated data are shown as the Mean ± SEM. (N=5 mice/group; **p*<0.05; ***p*<0.01; ****p*<0.001; *****p*<0.0001. Statistical analyses were performed by One-way ANOVA with post hoc testing). Exact *P* values are presented in Supplementary Data 1. Source data are provided as a Source Data file.

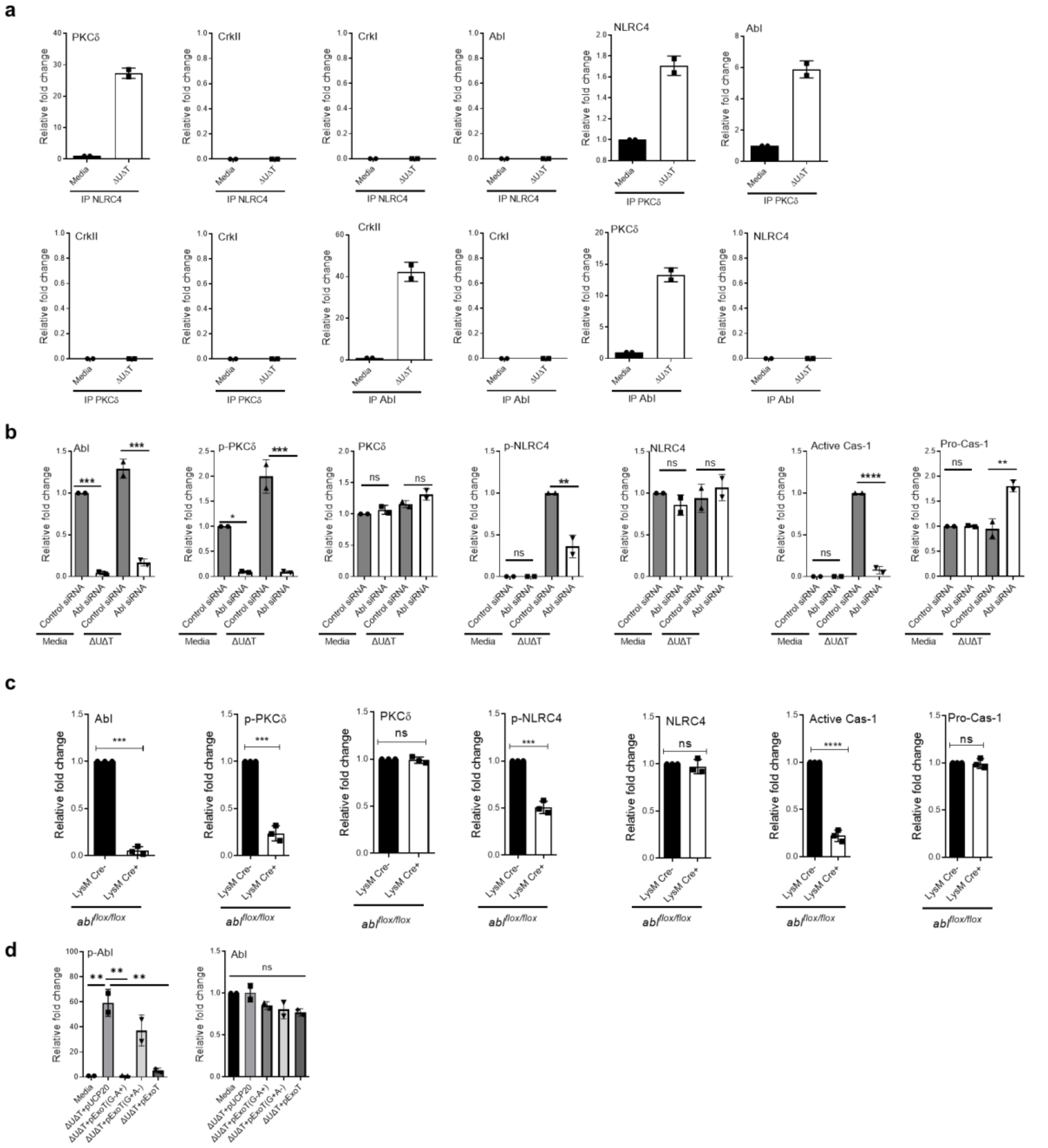

Supplementary Fig. 6. **Densitometer values associated with Fig. 6**. (a) Densitometer associated with Fig. 6a. (b) Densitometer associated with Fig. 6c. (c) Densitometer associated with Fig. 6f. (d) Densitometer associated with Figure 6i. Statistical analyses were determined by One-way ANOVA with post hoc testing for all except (c) which were done by two-sided unpaired Student's *t*-test). ns, Not Significant; **p*<0.05; ***p*<0.01; ****p*<0.001; *****p*<0.0001. Exact *P* values are presented in Supplementary Data 1. Source data are provided as a Source Data file.

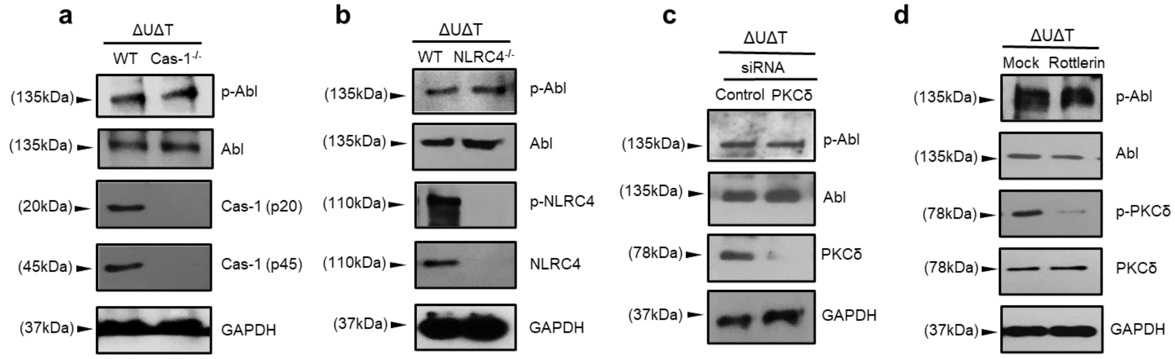

Supplementary Fig. 7. Phosphorylation cascade through NLRC4 inflammasome involves Abl/PKCδ/NLRC4 hierarchical order. (a-b) *Casp-1*$^{-/-}$ (a) and *Nlrc4*$^{-/-}$ (b) and C57BL/6 BMDMs (WT) were infected with indicated *P. aeruginosa* strain for 2h. Whole cell lysates were assessed for indicated proteins (phosphorylated and unphosphorylated forms) by Western blotting. (c) BMDMs from C57BL/6 were transfected for PKCδ siRNA for 48h. After transfection, cells were infected with indicated *P. aeruginosa* strain for 2h. Whole cell lysate was assessed for indicated proteins (phosphorylated and unphosphorylated forms) by Western blotting. (d) BMDMs from C57BL/6 were pretreated with PKC-δ inhibitor Rottlerin (5μM) for 90 min and then infected with ΔUΔT for 2h. Each experiment was repeated independently two times for a-b; and three times for c-d. Source data are provided as a Source Data file.

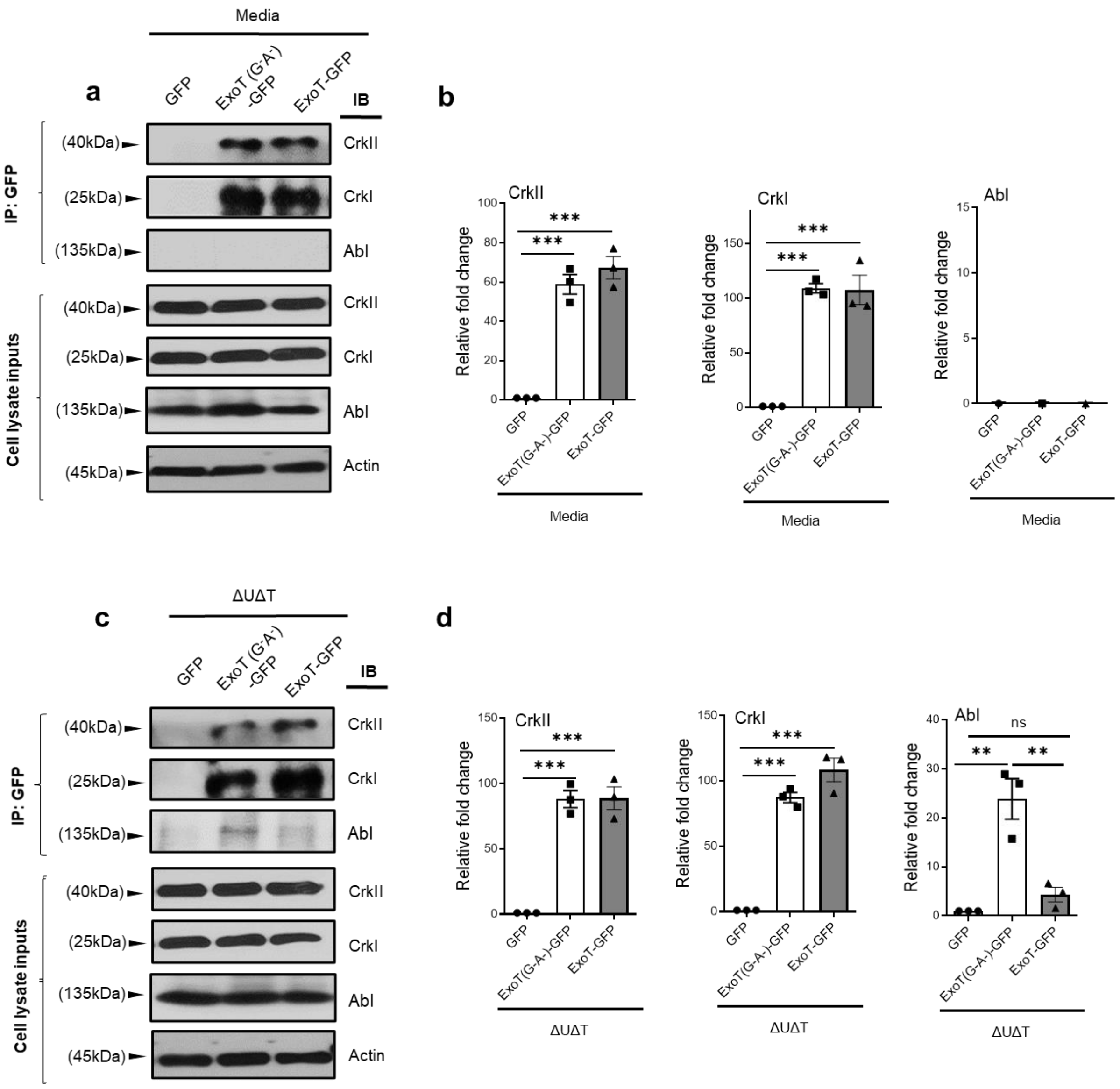

Supplementary Fig. 8. ExoT interaction analysis with Crk and Abl. (a-d) BMDMs were pretreated with a pan-caspase inhibitor (Z-VAD) at 60μM final concentration 1h prior to transfection with pExoT(G^-A^-)-GFP, pExoT-GFP, or pGFP expression vectors. 24h after transfection, cells were treated with PBS (a-b) or infected with indicated *P. aeruginosa* strain for 2h (c-d). Whole cell lysates were immunoprecipitated with antibodies against GFP and immunoblotted for indicated proteins (a & c). and the corresponding densitometer data are shown as the Mean ± SEM in (b & d). (N=3; ns, Not Significant; **p*<0.05; ***p*<0.01; ****p*<0.001; *****p*<0.0001. Statistical analyses were performed by One-way ANOVA with additional post hoc testing). Each experiment was repeated two times independently with N=2 replicates each time for

a&c and N=3 replicates each time for b&d. Exact *P* values are presented in Supplementary Data 1. Source data are provided as a Source Data file.

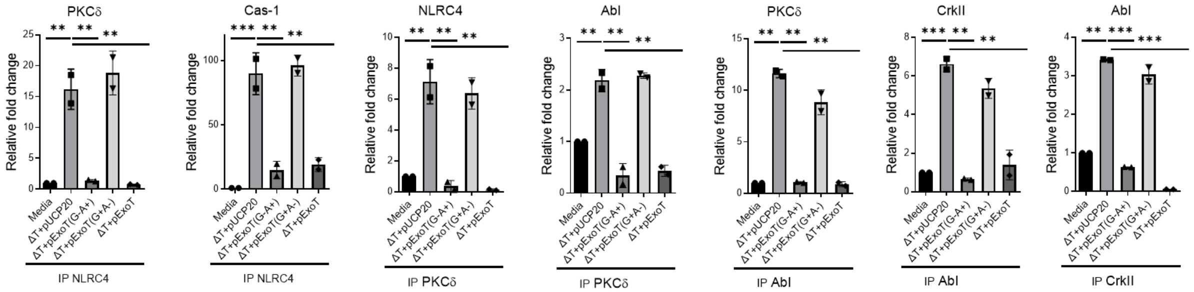

Supplementary Fig. 9. Data associated with Figure 6l. BMDMs from C57BL/6 were infected with indicated *P. aeruginosa* strains for 1h. Whole cell lysates and supernatants were immunoprecipitated with antibodies against NLRC4, PKCδ, Crk or Abl and immunoblotted for indicated proteins and the corresponding densitometer data are shown as the Mean ± SEM. (Statistical analyses were determined by One-way ANOVA with post hoc testing. Experiments were repeated twice independently. ns, Not Significant; *p<0.05; **p<0.01; ***p<0.001; ****p<0.0001). Exact P values are presented in Supplementary Data 1. Source data are provided as a Source Data file.

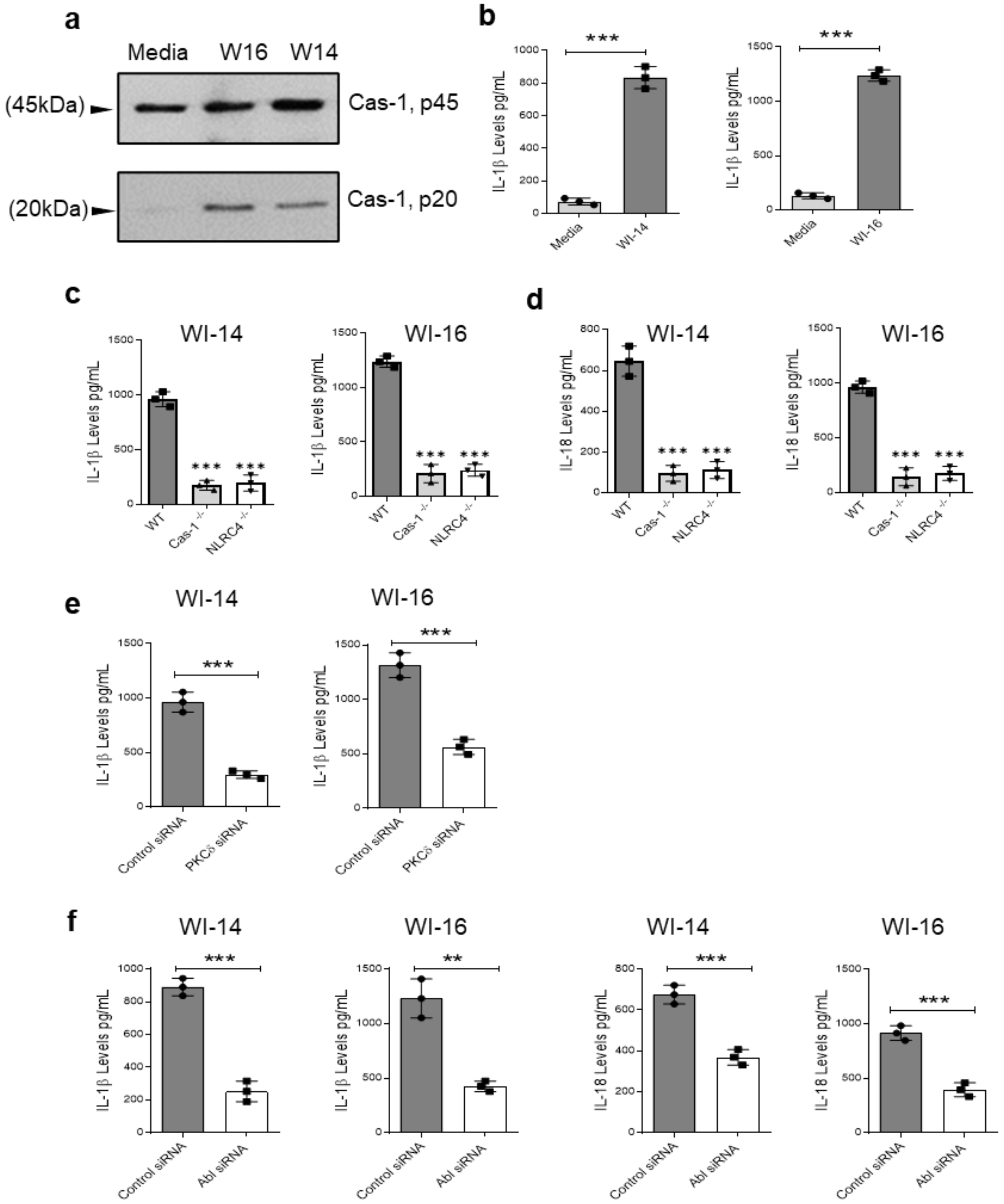

Supplementary Fig. 10. Abl and PKCδ functions are required for Caspase-1 inflammasome activation in BMDMs in response to *P. aeruginosa* wound clinical isolates. (a-b) BMDMs from C57BL/6 were infected with indicated *P. aeruginosa* clinical isolates for 2h and culture supernatants were assessed for caspase-1 activation by Western blotting (a) and for IL-1β and IL-18 cytokines by ELISA and the tabulated data are shown as the Mean ± SEM in (b) Experiments were repeated twice independently with N=2 replicates each time for a and N=3

replicates each time for (b). (c-d) *Casp-1*$^{-/-}$*, Nlrc4*$^{-/-}$ and C57BL/6 BMDMs were infected with indicated *P. aeruginosa* strain for 1h and culture supernatants were assessed for IL-1b and IL-18 cytokines by ELISA. (e-f) BMDMs from C57BL/6 were transfected for Abl or PKCδ siRNA for 24h and 48h respectively. After transfection, cells were infected with indicated bacterial strain for 2h. Culture supernatants were assessed for IL-1β and IL-18 cytokines by ELISA and the tabulated data are shown as the Mean ± SEM. (N=3; ns, Not Significant; *p<0.05; **p<0.01; ***p<0.001; ****p<0.0001. Statistical analyses were determined by two-sided unpaired Student's *t*-test for all except (c) which were done by One-way ANOVA with post hoc testing). Exact *P* values are presented in Supplementary Data 1. Source data are provided as a Source Data file.

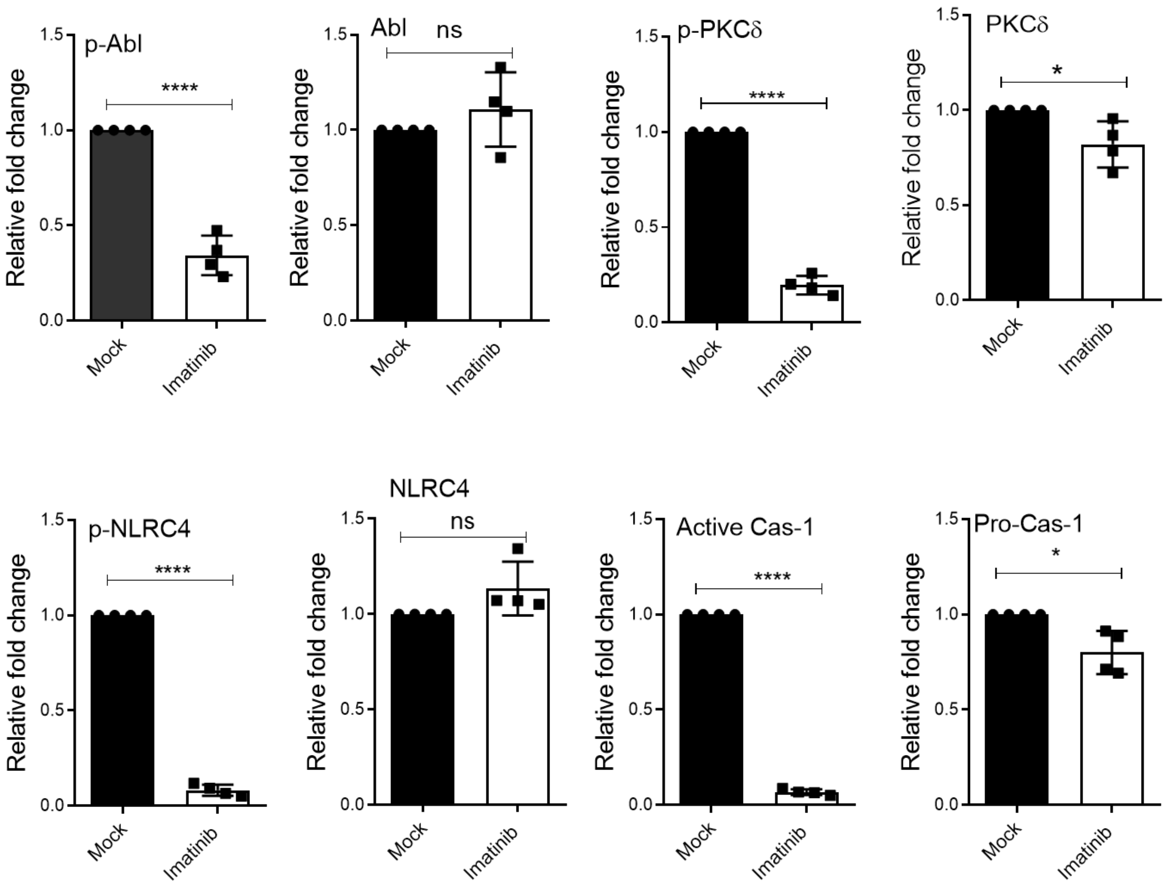

Supplementary Fig. 11. Densitometry data associated with Fig. 7c. C57bl/6 mice received Imatinib or saline by i.p. injection at 48, 24, and 2 hours before wounding and infection with 10^3 indicated *P. aeruginosa* ΔUΔT strain. Tissue homogenates were assessed for indicated proteins (phosphorylated and unphosphorylated forms) by Western blotting and the corresponding densitometer data are shown as the Mean ± SEM. (N=3; ns, Not Significant; **p*<0.05; ***p*<0.01; ****p*<0.001; *****p*<0.0001. Statistical analyses were determined by two-sided unpaired Student's *t*-test). Exact *P* values are presented in Supplementary Data 1. Source data are provided as a Source Data file.

Supplementary Table 1. Reagents and Resources

Antibodies and Reagents	SOURCE	IDENTIFIER
**Antibodies**		
ANTI-CASPASE-1 (P20)	AdipoGen	Cat. No. AG20B0042C100
ANTI-ASC PAB (AL177) 100 UG	AdipoGen	Cat. No. AG25B0006C100
Crk Mouse Monoclonal Antibody Clone: 22	BD	Cat. No. 610035
Ipaf (NLRC4) Rabbit anti-Human, Mouse, Polyclonal	Millipore Sigma	Cat. No. 06-112-5MI
Phospho-NLRC4 (Ser533) Mouse anti-Human, Mouse, Clone: 4B7B7	Invitrogen	Cat. No. PIMA531846
Goat anti-Mouse IgG (H+L) Cross-Adsorbed Secondary Antibody, Alexa Fluor 488	Invitrogen	Cat. No. A-11001
Goat anti-Rabbit IgG (H+L) Cross-Adsorbed Secondary Antibody, Texas Red	Invitrogen	Cat. No. T-6391
Polyclonal Antibody_PKC-delta Antibody	Cell Signaling Technology	Cat. No. 2058
Phospho-PKCδ (Tyr311) Antibody	Cell Signaling Technology	Cat. No. 2055
Mouse IL-1 beta /IL-1F2 Antibody	R &D systems	Cat. No. AF-401-NA
Mouse (MOPC-21) mAb IgG1 Isotype Control	Cell Signaling Technology	Cat. No. 4097
NLRC4 Polyclonal Antibody	Invitrogen	Cat. No. PA5-72908
NLRC4 Monoclonal Antibody	Abcam	Cat. No. ab201792
NLRC4 (SER-533) PAB	ECM Biosciences	Cat. No. NP5411
Anti- c-Abl (24-11)	Santa Cruz Biotechnology	Cat. No. sc-23
DONKEY ANTI-GOAT IGG) Secondary Antibody (HRP)	Novus Biologicals	Cat. No. NBP1-74815
β-Actin (13E5) Rabbit mAb	Cell Signaling Technology	Cat. No. 4970
Anti-Myeloperoxidase antibody	Abcam	Cat. No. ab9535
Anti-ABL1 (phospho Y245)	Abcam	Cat. No. ab193223
ANTI-RABBIT IGG, HRP linked Antibody	Cell Signaling Technology	Cat. No. 7074S
Anti-mouse IgG, HRP-linked Antibody	Cell Signaling Technology	Cat. No. 7076
GAPDH Antibody Rabbit Polyclonal	Proteintech	Cat. No. 1094-I-AP
Anti-horseradish peroxidase, Clone: HRPN	InVivoMab Antibodies	Cat. No. BE0088
**Reagents and kits**		
Hematoxylin	Thermo Fisher	Cat. No. 7111L
Eosin Y	Thermo Fisher	Cat. No. 7211L
Bluing Reagent	Thermo Fisher	Cat. No. 7301L
Gleevec (Imatinib mesylate)	Selleck Chemical LLC	Cat. No. 506719
Rottlerin	Acros Organics	Cat. No. AC328490100

PP2	Selleckchem	Cat. No. S7008
AZD0530	Selleckchem	Cat. No. S1006
FAK Inhibitor 14 (1,2,4,5-benzenetetraamine tetrahydrochloride)	Santa Cruz Biotechnology	Cat. No. SC-203950
Lyn peptide inhibitor	Tocris	Cat. No. 2265
TC-S 7003	Tocris	Cat. No. 3567/10
Ultrapure LPS, E. coli 0111: B4	InvivoGen	Cat. No. tlrl-3pelps
Direct IP Kit	Pierce	Cat. No. 26148
CRK I/II siRNA	Santa Cruz Biotechnology	Cat. No. SC-44854
Abl siRNA	Santa Cruz Biotechnology	Cat. No. SC-29844
PKCδ siRNA	Santa Cruz Biotechnology	Cat. No. SC-36246
Control siRNA	Santa Cruz Biotechnology	Cat. No. SC-37007
Lipofectamine RNAimax	Invitrogen	Cat. No. 13778075
FAM FLICA CASPASE 1 ASSAY KIT	Fisher Scientific	Cat. No.NC0585813
ProLong™ Gold Antifade Mountant with DAPI	Invitrogen	Cat. No. P36941
Z-VAD	R&D Systems	Cat. No. FMK001
**Critical Commercial Assays**		
IL-1β ELISA kit	Invitrogen	Cat. No. 501128814
IL-18 ELISA kit	Invitrogen	Cat. No. 501129088
**Experimental Models: Organisms**		
**Mouse**		
Mouse: C57BL/6J	Jackson laboratories	000664
Mouse: *NLRP1b*$^{-/-}$	Jackson laboratories	021301
Mouse: *NLRP3*$^{-/-}$	Jackson laboratories	021302
Mouse: *Cas-1*$^{-/-}$	Jackson laboratories	032662
Mouse: *Cas-11*$^{-/-}$	Jackson laboratories	024698
Mouse: *Pyrin*$^{+/-}$ (*Mefv*$^{+/-}$)	Jackson laboratories	021315
Mouse: *AIM2*$^{-/-}$	Jackson laboratories	013144
Mouse: *ASC*$^{-/-}$	Genentech	[120]
Mouse: *NLRC4*$^{-/-}$	Genentech	[72]
Mouse: *Abl*$^{flox/flox}$ *LysM Cre*+ mice		[97]
**BMDM**		
Mouse: *NLRC4*$^{-/-}$	Genentech	[72]
PA103	WT cytotoxic *P. aeruginosa* expressing ExoT and ExoU	[10,117]
T3SS^{-} (*pscJ::*Tn5)	Tn5 containing gentamycin inserted into *pscJ* gene. T3SS defective	[10,117]
ΔT	*P. aeruginosa* with an inframe deletion of ExoT	[11]

ΔT+pUCP20	*P. aeruginosa* with an inframe deletion of ExoT; complemented with vector only	This study
ΔT+pExoT	*P. aeruginosa* with an inframe deletion of ExoT; complemented with ExoT	This study
ΔU	*P. aeruginosa* with an inframe deletion of ExoU	[10]
ΔUΔT	*P. aeruginosa* with an inframe deletion of ExoU & ExoT	[10]
ΔUΔT+pUCP20	*P. aeruginosa* with an inframe deletion of ExoU & ExoT; complemented with vector only	[10]
ΔUΔT+pExoT	*P. aeruginosa* with an inframe deletion of ExoU & ExoT; complemented with ExoT	[10]
ΔUΔT+pExoT(G+A-)	*P. aeruginosa* with an inframe deletion of ExoU & ExoT; complemented with ExoT with a point mutation in ADPRT (EQE383-385AAA) domain of ExoT	[10]
ΔUΔT+pExoT(G-A+)	*P. aeruginosa* with an inframe deletion of ExoU & ExoT; complemented with ExoT with a point mutation in the GAP (R149K) domain of ExoT	[10]
**PAK strains**		
PAK	WT *P. aeruginosa* express ExoT, ExoS and ExoY	[13]
T3SS⁻ (*pscJ::*Tn5)	Tn5 containing gentamycin inserted into *pscJ* gene. T3SS defective	[13]
ΔT	*P. aeruginosa* with an inframe deletion of ExoT	[13]
ΔT+pUCP20	*P. aeruginosa* with an inframe deletion of ExoT; complemented with vector only	This study

ΔT+pExoT	*P. aeruginosa* with an inframe deletion of ExoT; complemented with ExoT	This study
ΔS	*P. aeruginosa* with an inframe deletion of ExoS	[13]
ΔSΔT	*P. aeruginosa* with an inframe deletion of ExoS & ExoT	[13]
ΔSΔT+pUCP20	*P. aeruginosa* with an inframe deletion of ExoS & ExoT; complemented with vector only	[13]
ΔSΔT+pExoT	*P. aeruginosa* with an inframe deletion of ExoS & ExoT; complemented with ExoT	[13]
WI-14	Wound clinical *P. aeruginosa* isolate	[105]
WI-16	Wound clinical *P. aeruginosa* isolate	[105]
**Plasmids**		
pUCP20	Broad-host-range cloning vector, IncP, Ap^r	[9]
pUCP20-ExoT	vector harboring functional ExoT, Ap^r	[9]
WT NLRC4	Genentech	[72]
NLRC4/S533A	Genentech	[72]
ExoT-GFP	pIRES mammalian expression vector expressing wild type ExoT, fused to GFP at the C-terminus	[10]
ExoT (G^-A^-)-GFP	pIRES mammalian expression vector expressing double mutant ExoT, fused to GFP at the C-terminus	[10]

pGFP	Vector control	[10]
**Software and Algorithms**		
GraphPad Prism	GraphPad	https://graphpad.com/scientific-software/prism/
