# Supplementary material for: CrkII/Abl phosphorylation cascade is critical for NLRC4 inflammasome activity and is blocked by Pseudomonas aeruginosa ExoT
Source: Nat Commun. 2022 Mar 11;13:1295. doi: 10.1038/s41467-022-28967-5 (PMC8917168; doi:10.1038/s41467-022-28967-5)
Supplement: Supplementary file 2 — Reporting Summary [file 41467_2022_28967_MOESM2_ESM.pdf]

nature research

Corresponding author(s): Sasha Shafikhani

Last updated by author(s): Feb 13, 2022

Reporting Summary

Nature Research wishes to improve the reproducibility of the work that we publish. This form provides structure for consistency and transparency in reporting. For further information on Nature Research policies, see our Editorial Policies and the Editorial Policy Checklist.

Statistics

For all statistical analyses, confirm that the following items are present in the figure legend, table legend, main text, or Methods section.

n/a	Confirmed	
☐	☒	The exact sample size (*n*) for each experimental group/condition, given as a discrete number and unit of measurement
☐	☒	A statement on whether measurements were taken from distinct samples or whether the same sample was measured repeatedly
☐	☒	The statistical test(s) used AND whether they are one- or two-sided<br>*Only common tests should be described solely by name; describe more complex techniques in the Methods section.*
☒	☐	A description of all covariates tested
☒	☐	A description of any assumptions or corrections, such as tests of normality and adjustment for multiple comparisons
☐	☒	A full description of the statistical parameters including central tendency (e.g. means) or other basic estimates (e.g. regression coefficient) AND variation (e.g. standard deviation) or associated estimates of uncertainty (e.g. confidence intervals)
☐	☒	For null hypothesis testing, the test statistic (e.g. *F*, *t*, *r*) with confidence intervals, effect sizes, degrees of freedom and *P* value noted<br>*Give P values as exact values whenever suitable.*
☒	☐	For Bayesian analysis, information on the choice of priors and Markov chain Monte Carlo settings
☒	☐	For hierarchical and complex designs, identification of the appropriate level for tests and full reporting of outcomes
☒	☐	Estimates of effect sizes (e.g. Cohen's *d*, Pearson's *r*), indicating how they were calculated

Our web collection on statistics for biologists contains articles on many of the points above.

Software and code

Policy information about availability of computer code

Data collection	Imaging data were collected on Axiovert Z1 microscope (ZEISS), using ZEISS ZEN blue edition software.
Data analysis	We used GraphPad Prism version 8 (GraphPad software), Excel (Microsoft Office, version, Professional Plus 2016), Image J (open source, version, 1.52a), and Illustrator 2021 (Adobe, version, 25.4.3), to analyze the data in this study.

For manuscripts utilizing custom algorithms or software that are central to the research but not yet described in published literature, software must be made available to editors and reviewers. We strongly encourage code deposition in a community repository (e.g. GitHub). See the Nature Research guidelines for submitting code & software for further information.

Data

Policy information about availability of data

All manuscripts must include a data availability statement. This statement should provide the following information, where applicable:

- Accession codes, unique identifiers, or web links for publicly available datasets
- A list of figures that have associated raw data
- A description of any restrictions on data availability

The main data supporting the findings of this study are available within the article and its Supplementary Figures. The source data underlying all Figures and Supplementary Figures are provided as a Source Data file. Source Data file provided with this paper.

Field-specific reporting

Please select the one below that is the best fit for your research. If you are not sure, read the appropriate sections before making your selection.

☒ Life sciences ☐ Behavioural & social sciences ☐ Ecological, evolutionary & environmental sciences

For a reference copy of the document with all sections, see nature.com/documents/nr-reporting-summary-flat.pdf

Life sciences study design

All studies must disclose on these points even when the disclosure is negative.

Sample size	Sample size was determined based on our previous experience (references, 12,56,59,116,119 and was large enough to detect differences between samples with biological significance.<br>Reference:<br>Gupta, K. H. et al. Apoptosis and Compensatory Proliferation Signaling Are Coupled by CrkI-Containing Microvesicles. Dev Cell 41, 674-684 e675, doi:10.1016/j.devcel.2017.05.014 (2017).<br><br>In general, no calculations were done to determine sample size. Sample size was determined based on standards for experimental cell biology and animal studies, attempting to have a minimum of N = 3 biological replicates with sufficient reproducibility. Exceptions included experiments where an extraordinary large sample size was required. Regarding cell<br>numbers per experiment, the parameters employed were such that healthy cell cultures were maintained and studied, while providing enough material for protein levels analysis. In<br>experiments involving animals, N = 4-8 with further experimental details on sample analysis are included in the<br>figure legends.
Data exclusions	No data were excluded from these studies
Replication	Results were verified by independent biological and technical repeats as described in the figure legends. Attempts of data replication were, therefore successful.
Randomization	Animals were randomized, and assigned to different groups by the investigator. Gender and age were matched across groups.
Blinding	No blinding was used as the investigator performed all experiments.

Reporting for specific materials, systems and methods

We require information from authors about some types of materials, experimental systems and methods used in many studies. Here, indicate whether each material, system or method listed is relevant to your study. If you are not sure if a list item applies to your research, read the appropriate section before selecting a response.

Materials & experimental systems

n/a	Involved in the study
☐	☒ Antibodies
☒	☐ Eukaryotic cell lines
☒	☐ Palaeontology and archaeology
☐	☒ Animals and other organisms
☒	☐ Human research participants
☒	☐ Clinical data
☒	☐ Dual use research of concern

Methods

n/a	Involved in the study
☒	☐ ChIP-seq
☒	☐ Flow cytometry
☒	☐ MRI-based neuroimaging

Antibodies

Antibodies used	Antibodies: Anti-caspase-1 (P20) (AdipoGen Cat. No. AG20B0042C100, 1:1,000), anti-ASC pab (AL177) (AdipoGen Cat. No. AG25B0006C100, 1:1,000), Crk Mouse Monoclonal Antibody Clone: 22 (BD Cat. No. 610035, 1:1,000), NLRC4 (Abcam Cat. No. ab201792 and Millipore Sigma Cat. No. 06-112-5MI, 1:200), Phospho-NLRC4 (SER-533) PAB (ECM Biosciences Cat. No. NP5411, 1:50), Phospho-NLRC4 (Ser533) Mouse anti-Human, Mouse, Clone: 4B7B7 (Invitrogen Cat. No. PIMA531846, 1:50), Polyclonal Anti-PKCd (Cell Signaling Technology Cat. No. 2058, 1:1,000), Phospho-PKCd(Tyr311) Antibody (Cell Signaling Technology Cat. No. 2055, 1:500), Mouse IL-1β Antibody (R&D systems Cat. No. AF-401-NA, 1:1,000). Mouse (MOPC-21) mAb IgG1 Isotype Control (Cell Signaling Technology, Cat. No. 4097, 1:1,000) NLRC4 Polyclonal Antibody Invitrogen (Cat. No. PA5-72908, 1:100), Anti- c-Abl (24-11,) (Santa Cruz Biotechnology Cat. No. sc-23, 1:100), donkey anti-goat IGG Secondary Antibody (HRP) (Novus Biologicals Cat. No. NBP1-74815, 1:1,000), β-Actin (13E5) Rabbit mAb (Cell Signaling Technology, Cat. No. 4970, 1:2,000) Anti-Myeloperoxidase antibody (Abcam Cat. No. ab9535, 1:1,000), Anti-ABL1 (phosphor-Y245) (Abcam Cat. No. ab193223, 1:100), anti-rabbit IGG, HRP linked Antibody (Cell Signaling Technology, Cat. No. 7074S, 1:5,000), Anti-mouse IgG, HRP-linked Antibody (Cell Signaling Technology, Cat. No. 7076,

1:5,000), GAPDH Antibody Rabbit Polyclonal (Proteintech Cat. No. 1094-I-AP, 1:5,000).
immunoprecipitated with antibodies against NLRC4 (NLRC4 Polyclonal Antibody (Invitrogen Cat. No. PA5-72908 and Abcam Cat. No. ab201792, 1:50)), PKCδ (Cell Signaling Technology, Cat. No. 2058, 1:50) and Crk (BD Cat. No. 610035, 1:50), and Abl (Santa Cruz Biotechnology Cat. No. sc-23, 1:50) antibodies against Flag (Millipore Sigma, Cat. No. F1804, 1:50). antibodies against GFP (Abcam, Cat. No. AB5450, 1:50)
Fluorescence Microscopy
antibodies against ASC (AdipoGen Cat. No. AG25B0006C100, 1:200), caspase-1 (AdipoGen Cat. No. AG20B0042C100, 1:200), and p-NLRC4 (Invitrogen Cat. No. PIMA531846, 1:100)
Goat anti-Mouse IgG (H+L) Secondary Antibody, Alexa Fluor 488 (Invitrogen Cat. No. A-11001, 1:500), Goat anti-Rabbit IgG (H+L) Secondary Antibody, Texas Red (Invitrogen Cat. No. T-6391, 1:500), at 1:500 dilution

Validation

All antibodies in this study were published before and were highly cited in many journals. Antibodies were validated based on the manufacturer's website, which includes relevant literature (for catalogue number and company information, please see above and also Supplementary table1). Antibodies were validated also in Manuscript by comparing wildtype and knockout mice (please see figures and supplemental figures).

anti -caspase -1 (AdipoGe n, AG-20B-0042): Measuring the inflammasome: 0. Gross; Methods Mol. Biol. (2012). https :// adipogen .com/ ag -20 b-0042-anti -caspase-1- p20-mouse-ma b-casper-1.html

anti-ASC (AdipoGen, AG-25B-006-Cl00): Human NLRPl is a sensor for double-st randed RNA. S. Bauernfried; Science (2021). http s://a dipogen.com/ag -25 b-0006-a nti -asc- pab-al177.html/

Crk Mouse Monoclonal Antibody Clone: 22 (BD Cat. No. 610035, 1:1,000), Cho SY, Klemke RL. Purification of pseudopodia from polarized cells reveals redistribution and activation of Rac through assembly of a CAS/Crk scaffold. J Cell Biol. 2002; 156(4):725-736. (Clone-specific: Immunoprecipitation, Western blot).https://www.bdbiosciences.com/ja-jp/products/reagents/microscopy-imaging-reagents/immunofluorescence-reagents/purified-mouse-anti-crk.610035

NNLRC4 (Abcam Cat. No. ab201792 1:200, Wang W et al. Galectin-9 Targets NLRP3 for Autophagic Degradation to Limit Inflammation. J Immunol 206:2692-2699 (2021).https://www.abcam.com/nlrc4-antibody-epr19733-ab201792.html

NLRC4 (Millipore Sigma Cat. No. 06-112-5MI, 1:200), An activating NLRC4 inflammasome mutation causes autoinflammation with recurrent macrophage activation syndrome. Canna, SW; de Jesus, AA; Gouni, S; Brooks, SR; Marrero, B; Liu, Y; DiMattia, MA; Zaal, KJ; Sanchez, GA; Kim, H; Chapelle, D; Plass, N; Huang, Y; Villarino, AV; Biancotto, A; Fleisher, TA; Duncan, JA; O'Shea, JJ; Benseler, S; Grom, A; Deng, Z; Laxer, RM; Goldbach-Mansky, R Nature genetics 46 1140-6 2014, https://www.emdmillipore.com/US/en/product/Anti-Ipaf-NLRC4-Antibody,MM_NF-06-1125#documentation.

Phospho-NLRC4 (SER-533) PAB (ECM Biosciences Cat. No. NP5411, 1:50), Qu, Y. et al. (2012) Nature 490:539.
Elinav, E. et al. (2011) Immunity 34:665.https://ecmbio.com/products/np5411

Phospho-NLRC4 (Ser533) Mouse anti-Human, Mouse, Clone: 4B7B7 (Invitrogen Cat. No. PIMA531846, 1:50)Species Reactivity Human, Mouse, Mouse / IgG2b, Monoclonal, Antibody Clone 4B7B7, https://www.thermofisher.com/antibody/product/Phospho-NLRC4-Ser533-Antibody-clone-4B7B7-Monoclonal/MA5-31846.

Polyclonal Anti-PKCd (Cell Signaling Technology Cat. No. 2058, 1:1,000) Nikos Koundouros, et. al. Metabolic Fingerprinting Links Oncogenic PIK3CA with Enhanced Arachidonic Acid-Derived Eicosanoids. Cell,
2020, https://www.cellsignal.com/products/primary-antibodies/pkcd-antibody/2058

Phospho-PKCdTyr311) Antibody (Cell Signaling Technology Cat. No. 2055, 1:500), Yoshiaki Kitamura, et. al. Effects of corticosteroid on mRNA levels of histamine H1 receptor in nasal mucosa of healthy participants and HeLa cells. J Med Invest, 2020, https://www.cellsignal.com/products/primary-antibodies/phospho-pkcdelta-tyr311-antibody/2055

Mouse IL-1β Antibody (R&D systems Cat. No. AF-401-NA, 1:1,000). Terminal uridyltransferase 7 regulates TLR4-triggered inflammation by controlling Regnase-1 mRNA uridylation and degradation
Authors: CC Lin, YR Shen, CC Chang, XY Guo, YY Young, TY Lai, IS Yu, CY Lee, TH Chuang, HY Tsai, LC Hsu
Nature Communications, 2021;12(1):3878.https://www.rndsystems.com/products/mouse-il-1beta-il-1f2-antibody_af-401-na#product-citations

Mouse (MOPC-21) mAb IgG1 Isotype Control (Cell Signaling Technology, Cat. No. 4097, 1:1,000), BoA Kim, et. al. Endothelial pyruvate kinase M2 maintains vascular integrity. J Clin Invest, 2018. https://www.cellsignal.com/products/antibody-conjugates/mouse-mopc-21-mab-igg1-isotype-control-biotinylated/4097

NLRC4 Polyclonal Antibody Invitrogen (Cat. No. PA5-72908, 1:100), https://www.thermofisher.com/antibody/product/NLRC4-Antibody-Polyclonal/PA5-72908

Anti- c-Abl (24-11,) (Santa Cruz Biotechnology Cat. No. sc-23, 1:100), PMID: # 33777928 Montecino, F.|González, N.|Blanco, N.|Ramírez, MJ.|González-Martín, A.|Alvarez, AR.|Olguín, H.| et al. 2021. Front Cell Dev Biol. 9: 606403.https://www.scbt.com/p/c-abl-antibody-24-11

donkey anti-goat IGG Secondary Antibody (HRP) (Novus Biologicals Cat. No. NBP1-74815, 1:1,000),
Perell GT, Mishra NK, Sudhamalla B et al. Specific Acetylation Patterns of H2A Z Form Transient Interactions with the BPTF Bromodomain. Biochemistry. 2017 Sep 05 [PMID: 28771339] (WB), https://www.novusbio.com/products/igg-h-l-antibody_nbp1-74811#reviews-publications

β-Actin (13E5) Rabbit mAb (Cell Signaling Technology, Cat. No. 4970, 1:2,000)

Yun Yang, et. al.Altered succinylation of mitochondrial proteins, APP and tau in Alzheimer's disease.Nat Commun, 2022, https://www.cellsignal.com/products/primary-antibodies/b-actin-13e5-rabbit-mab/4970

Anti-Myeloperoxidase antibody (Abcam Cat. No. ab9535, 1:1,000), Riffelmacher T et al. Metabolic activation and colitis pathogenesis is prevented by lymphotoxin ß receptor expression in neutrophils. Mucosal Immunol 14:679-690 (2021). https://www.abcam.com/myeloperoxidase-antibody-ab9535.html

Anti-ABL1 (phosphor-Y245) (Abcam Cat. No. ab193223, 1:100),Zhou HJ et al. Caveolae-mediated Tie2 signaling contributes to CCM pathogenesis in a brain endothelial cell-specific Pdcd10-deficient mouse model. Nat Commun 12:504 (2021).https://www.abcam.com/abl1-phospho-y412-antibody-ab4717.html

GAPDH Antibody Rabbit Polyclonal (Proteintech Cat. No. 1094-I-AP, 1:5,000). Li YanA , CREB1/miR-433 reciprocal feedback loop modulates proliferation and metastasis in colorectal cancer.Aging (Albany NY), 2018, https://www.ptglab.com/products/GAPDH-Antibody-10494-1-AP.htm#publications

antibodies against Flag (Millipore Sigma, Cat. No. F1804, 1:50).Monika Srivastava et al. Roquin binds microRNA-146a and Argonaute2 to regulate microRNA homeostasis.Nature communications, 6, 6253-6253 (2015-02-24)

antibodies against GFP (Abcam, Cat. No. AB5450, 1:50) Pavel M et al. a-Catenin levels determine direction of YAP/TAZ response to autophagy perturbation. Nat Commun 12:1703 (2021). https://www.abcam.com/gfp-antibody-ab5450.html.

Animals and other organisms

Policy information about studies involving animals; ARRIVE guidelines recommended for reporting animal research

Laboratory animals	Mouse: C57BL/6J Jackson laboratories 000664<br>Mouse: NLRP1b-/- Jackson laboratories 021301<br>Mouse: NLRP3-/- Jackson laboratories 021302<br>Mouse: Cas-1-/- Jackson laboratories 032662<br>Mouse: Cas-11-/- Jackson laboratories 024698<br>Mouse: Pyrin+/- (Mefv+/−) Jackson laboratories 021315<br>Mouse: AIM2-/- Jackson laboratories 013144<br>Mouse: ASC-/- Genentech N/A<br>Mouse: NLRC4-/- Genentech N/A<br>Abl flox/flox LysM Cre+<br><br>Males and females, 7-8 week old mice.
Wild animals	No wild animals were used in this study.
Field-collected samples	The study did not involve samples collected from the field.
Ethics oversight	We have approval from Rush Institutional Animal Care and Use Committee (IACUC No: 18-013) to conduct research as indicated. All procedures complied strictly with the standards for care and use of animal subjects as stated in the Guide for the Care and Use of Laboratory Animals (Institute of Laboratory Animal Resources, National Academy of Sciences, Bethesda, MD, USA).

Note that full information on the approval of the study protocol must also be provided in the manuscript.
