# Supplementary material for: CrkII/Abl phosphorylation cascade is critical for NLRC4 inflammasome activity and is blocked by Pseudomonas aeruginosa ExoT
Source: Nat Commun. 2022 Mar 11;13:1295. doi: 10.1038/s41467-022-28967-5 (PMC8917168; doi:10.1038/s41467-022-28967-5)
Supplement: Supplementary file 3 — Description of Additional Supplementary Files [file 41467_2022_28967_MOESM3_ESM.pdf]

Title: Supplementary Data 1.

Description: Statistical analysis and exact *P*-values for all figures and supplementary figures in the manuscript. Statistical analyses between two groups were performed by two-sided unpaired Student's *t*-test and statistical analyses of more than two groups were performed by One-way analysis of variance (ANOVA) with additional post hoc testing, using the GraphPad Prism software, version 8. *P*-values less than or equal to 0.05 were taken as significant. (ns, Not Significant; *$p<0.05$; **$p<0.01$; ***$p<0.001$; ****$p<0.0001$).
